# New Horizons in Skin Sensitization Assessment of Complex Mixtures: The Use of New Approach Methodologies Beyond Regulatory Approaches

**DOI:** 10.3390/toxics13080693

**Published:** 2025-08-20

**Authors:** Argel Islas-Robles, Meera Ramani, Jakeb Phillips, Gertrude-Emilia Costin

**Affiliations:** Department of Skin Sensitization, Institute for In Vitro Sciences, Inc. (IIVS), Gaithersburg, MD 20878, USA

**Keywords:** allergic contact dermatitis, skin sensitization, New Approach Methodologies (NAMs), botanicals, extracts, medical devices, mixtures, wearables, pollutants, in vitro, in chemico, in silico

## Abstract

Skin sensitization is a complex biological process induced by a wide range of chemicals, from single molecules to complex mixtures and finished products. While single chemical entities were used to design and validate sophisticated safety assessment assays, complex chemistries have proven challenging to test in practice using these methods. These assays range from in silico and in chemico methods to cell-based and reconstructed tissues-based approaches and target the key events now grouped within the Adverse Outcome Pathway. We focused our analysis on the use of New Approach Methodologies for skin sensitization assessments of complex mixtures, botanicals, medical and wearable devices, agrochemicals, and pollutants. We present the defined approaches that integrate these technologies, aligning with the principles of the replacement, reduction, and refinement of animal testing. We also detail the known challenges posed by these product classes in terms of testing and data interpretation. Our analysis indicates that validated and non-validated NAMs have shown some success in predicting skin sensitization potential across the product categories reviewed.

## 1. Introduction

Allergic contact dermatitis (ACD) presents clinically as a skin inflammatory process that occurs as a result of direct contact and interaction between the skin and allergens. ACD is a cell-mediated, type IV hypersensitivity reaction conditioned by prior exposure to allergens [1], with a prevalence of 20.1% in the general population [2]. The likelihood of developing ACD depends not only on the inherent sensitizing potential (hazard) of the substance but also on the extent and frequency of exposure, which together define the overall risk [3]. Besides metals, which were identified early as ACD inducers, the second largest cause is exposure to cosmetic products, particularly if fragrances and preservatives are included in their composition [2]. Beyond cosmetics, other product categories have also raised concerns regarding skin sensitization potential, underscoring the need for tailored testing strategies that prioritize human-relevant approaches. For example, essential oils, botanical ingredients, and plant extracts, which are used by the cosmetic, personal care, and other industries, are complex mixtures with variable chemical components that may cause ACD [4]. Extending beyond cosmetics, agrochemical formulations, which often contain multiple active and inert ingredients, pose particular challenges for skin sensitization assessment, prompting recent efforts to evaluate the applicability of non-animal defined approaches for their hazard identification [5]. Medical devices have also recently gained attention for the application of non-animal methods in skin sensitization assessment. These include a wide array of items used in diagnosis, treatment, or disease prevention, all of which require rigorous biocompatibility evaluations to ensure patient safety. Although New Approach Methodologies (NAMs) have been successfully applied in other sectors, their use in medical device testing remains limited due to regulatory hesitation and the need for validation tailored to the unique properties of device materials [6]. Similarly, wearable devices such as fitness trackers, smartwatches, and personal care accessories represent an emerging category of interest, as they are designed for continuous or repeated skin contact and may pose sensitization risks due to leachable substances [7].

The complexity of products that come into contact with human skin has increased significantly over the years, in parallel with the consumers’ demands for safe, efficacious, organic, and natural goods, advancements in health-related technologies, and the widespread use of agrochemicals in the food industry and environmental systems. The reaction to allergens is often a reflection of minimally or uncharacterized newly emerged categories of substances that might be associated with innovation or perceived added appealing properties [8].

Besides single chemical entities used to evaluate the testing methods (e.g., small organic molecules or easily dissolved chemicals), the chemical industry manufactures a considerably large portfolio of complex mixtures and finished products. These are broadly categorized as Botanical or Natural Substances (BNSs) and Unknown or Variable Composition, Complex Reaction Products or Biological Materials (UVCBs). BNSs are defined as naturally occurring complex mixtures from plants or living/previously living organisms [9]. The self-explanatory complexity of UVCBs makes them very challenging to characterize in terms of identifying, with precision and accuracy, each component and its respective concentration or interactions/synergistic effects. To add to the current complex landscape, UVCBs account for up to 20% of recent registrations in the European Union under the regulation on the Registration, Evaluation, and Authorization of Chemicals (REACH) and represent a significant part of cosmetic components [10].

Although they are classified within the wide groups of BNSs and UVCBs, the categories of interest for our analysis are basically various types of mixtures. It is therefore important to provide an accurate definition of what a mixture represents. The US Environmental Protection Agency (EPA) defines chemical mixtures as either (a) simple mixtures, containing “two or more identifiable components but few enough that the mixtures’ toxicity can be adequately characterized by a combination of the components’ toxicities and the components’ interactions”; or (b) complex mixtures containing “so many components that any estimation of their toxicity based on their components’ toxicities contains too much uncertainty and error to be useful” [11]. Furthermore, according to the Organization for Economic Cooperation and Development (OECD), a mixture is composed of two or more substances that do not react chemically. If one main constituent is present to at least 80% (*w*/*w*), even after accounting for impurities [12], the substance is called mono-constituent; if more than one main constituent is present in a concentration ≥10% (*w*/*w*) and <80% (*w*/*w*), the substance is defined as multi-constituent. The difference is that a mixture is obtained by blending without chemical reaction, while a multi-constituent substance is the result of a chemical reaction [13].

From a composition’s complexity perspective, BNSs and UVCBs are challenging for regulatory decision-making because very few established frameworks exist for their assessment [10]. While NAMs have been evaluated regarding their performance to address skin sensitization [14,15,16,17,18,19] and have been included in Defined Approaches (DAs) that are currently in official use [20], they are based mainly on the testing of individual or discrete chemicals. From the practical perspective of testing, these are considered “easy-to-test” substances. The reality within manufacturing industries, whether cosmetic, chemical, or otherwise, is that they routinely work with multi-constituent substances and finished products that are often considered “difficult-to-test” from a toxicological assessment perspective. Besides UVCBs, included in this category are also poorly water-soluble (<60 mg/L) chemicals and surfactants [16]. Therefore, BNSs and UVCBs are considered to be outside of some of the methods’ applicability domains or current established prediction models. Under the circumstances, a positive result obtained by testing may be informative and could indicate some sensitization potential, but a negative result is typically considered inconclusive.

Our analysis is focused on the following categories of BNS, UVCBs, or generally difficult-to-test products: botanicals, medical and wearable devices, agrochemicals, pollutants, and other uniquely designed complex mixtures. The investigation is restricted to these categories because they are representative products under the purview of the United States Food and Drug Administration (US FDA) (medical devices) and under the jurisdiction of the US EPA (agrochemicals), or they are not strictly regulated (botanicals and wearables). The majority of the other mixtures analyzed were used as tools to evaluate the sensitivity of the NAMs of interest or to calibrate them to the performance of animal or human clinical tests used as references. These mixtures were also used to mimic possible combinations of skin sensitizers that could occur in topically applied cosmetics, personal care products, or other classes of finished products. Lastly, we identified air pollutants as a rather unique category of UVCBs that is yet to be explored. This uncharted territory is wide open to the vast possibilities provided by NAMs and the modern scientific landscape, which together support complex testing and research programs. We present herein the testing strategies that have shown varying degrees of success for these challenging mixtures and finished formulations and the interpretations of their skin sensitization potential using both regulatory and non-regulatory NAMs.

The objective of this review is to critically assess the applicability and limitations of NAMs, both validated and non-validated, for evaluating the skin sensitization potential of complex mixtures and finished products within the categories mentioned before. By focusing on these product classes, we aim to provide clarity on the performance of existing NAMs, identify common challenges, and highlight opportunities for refining testing strategies. The use of NAMs in this context is also discussed in relation to regulatory frameworks and as part of a broader effort to promote, advance, and comply with the principles of the replacement, reduction, and refinement of animal testing (3Rs) [21].

## 2. Landscape for the Evaluation of Skin Sensitization Potential

### 2.1. Animal Testing and Human Clinical Studies

The evaluation of the skin sensitization potential of ingredients or finished formulations is an integral part of the continuous development of new or improved consumer product lines. This can be accomplished by risk assessment, which is a multi-step iterative process involving pre-clinical and clinical evaluation [22]. Within this process, the skin sensitization potential is evaluated based on available analytical and structural characterization followed by literature reviews and pre-clinical testing as needed (in vivo or NAMs-based in more recent years).

The assessment of skin sensitization potential has been historically conducted using methods based on laboratory animals. The established methods are the Guinea Pig Maximization Test (GPMT), the Buehler Test (OECD TG 406) [23], or murine tests, such as the Local Lymph Node Assay (LLNA, OECD TG 429) [24]. The LLNA includes three non-radioactive modifications: Daicel Chemical Industries (DA) (OECD TG 442A) [25,26] and two 5-bromo-2-deoxyuridine (BrdU) methods: an enzyme-linked immunosorbent assay (ELISA) and a flow cytometry method (FCM) (OECD TG 442B) [27]. The GPMT and Buehler Test assess the induction and elicitation phases of the skin sensitization pathway, while the murine tests evaluate the induction response. The results of the LLNA are reported as the estimated concentration, in weight percent, of a substance required to produce a 3-fold stimulation of lymphocyte proliferation in the draining lymph node compared to the solvent controls (EC3 values). The LLNA is regarded as a refined method that supports the reduction in animal use in testing, in line with the 3Rs principles.

Although animal tests such as the GPMT and the LLNA have contributed valuable insights into skin sensitization potential identification, these models are also associated with challenges in reproducibility, sensitivity, and relevance to human biology. For instance, the intra-laboratory repeatability of the LLNA and GPMT has been reported as 89% and 93%, respectively. However, when comparing classifications across species, concordance between the LLNA and GPMT models for a set of 403 substances was found to be only 77%, highlighting inconsistencies in hazard identification even among traditional assays [28]. These limitations have further underscored the need for more human-relevant, reproducible, and ethically sound approaches.

Our analysis included multiple studies that evaluated a variety of NAMs in comparison with paired in vivo data. The LLNA provided the vast majority of the data sets either as the only resource or in combination with GPMT and/or with human clinical data.

The pre-clinical evaluation informs the need to conduct subsequent clinical studies and guides product design to ensure that humans will not experience adverse effects when coming in contact with topically applied substances. Data obtained in human predictive patch tests (HPPTs) can be used to determine the skin sensitization potential [29]. This testing methodology has evolved over more than 50 years since first proposed in 1944 by Schwartz and Peck [30] and has since been extensively reviewed [31,32,33,34]. The most frequently used tests are the Human Maximization Test (HMT) and the Human Repeat Insult Patch Test (HRIPT). Both are based on the repeated exposure of individuals without known allergies to a potential skin sensitizer over the course of several weeks (induction phase). After a rest period, a challenge phase is conducted where the substance is reapplied to determine whether an allergic response is elicited (elicitation phase). The fundamental difference between these two tests is that the HMT is conducted on a smaller number of subjects and incorporates pre-treatment with an irritant to enhance skin penetration. Of the two tests, HRIPT is the most reliable and is frequently reported to be able to confirm a No Observed Effect Level (NOEL). It is also used to establish a No Expected Sensitization Induction Level (NESIL) within the Quantitative Risk Assessment (QRA) framework [35] or to demonstrate that humans will not respond adversely to a particular formulation. More recently, a Next Generation Risk Assessment (NGRA) framework has been established in which the HRIPT is used as a tool to confirm the absence of skin sensitization potential identified through NAMs. In this approach, the HRIPT is viewed as an acceptable human-based test that, together with NAMs, supports a strategy that eliminates reliance on animal testing and its associated ethical concerns [36]. The test also allows the detection of pre-existing skin sensitization to test materials, as confirmed by persistent skin reactions identified early in the induction period. As part of the risk assessment process, the HRIPT is a confirmatory method and is generally not used for the determination of skin sensitization hazard. However, if conducted with an appropriate number of subjects, the method is considered to be sensitive enough to detect induced skin sensitization when exposure to sensitizers is sufficiently high [22,37].

In general, even though the phases used in HPPTs are the same, variations still exist regarding the patch type, number of subjects, skin site exposed, number of induction patches, patch application time, duration, and rest period prior to challenge. For all methods, the assessment of skin sensitization is based on whether the skin response observed following the challenge application is greater than the reactions seen during the initial induction phase, indicating a memory-type immune response, which is consistent with sensitization. Occasionally, HPPT procedures may need to be modified to address specific test substance characteristics; for example, by using semi-occlusive patches for products that are too irritant to be tested under full occlusion. Therefore, these modifications need to be clearly reported, as they are taken into account in subsequent data interpretations, especially when human data are used to calibrate the performance of NAMs for skin sensitization.

HPPTs differ from diagnostic patch tests, which are performed on patients to identify the chemical source of ACD but do not provide information on previous exposure [38]. Other complementary but less reliable sources of human data may be clinical in-use testing and post-market monitoring/follow-up of consumer comments.

Most product categories analyzed in this review included HRIPT or HMT data; however, the source and quality of exposure data were often unclear, complicating comparisons with animal or NAM data. Thus, given the customization for clinical studies, it is important to specify the exact conditions in which the testing has been conducted in order to allow further comparative analysis.

### 2.2. Modern Established NAMs and Emerging Testing Trends

Following the adoption of REACH and of the seventh amendment to the EU Cosmetics Directive that banned from the European market cosmetic products containing ingredients tested on animals, a wide variety of alternative methods to assess skin sensitization have been developed, primarily focused on discrete chemicals [39]. They vary in complexity from in chemico to cell- and reconstructed tissues- or explants-based methodologies that can provide mechanistic insights. These assays are expanding in use, from cosmetic ingredients to finished products and, more recently, to the assessment of device extracts. However, a major challenge identified when using these methods is represented by the technical difficulties encountered when assessing the sensitization potential of complex mixtures.

**Figure 1:**
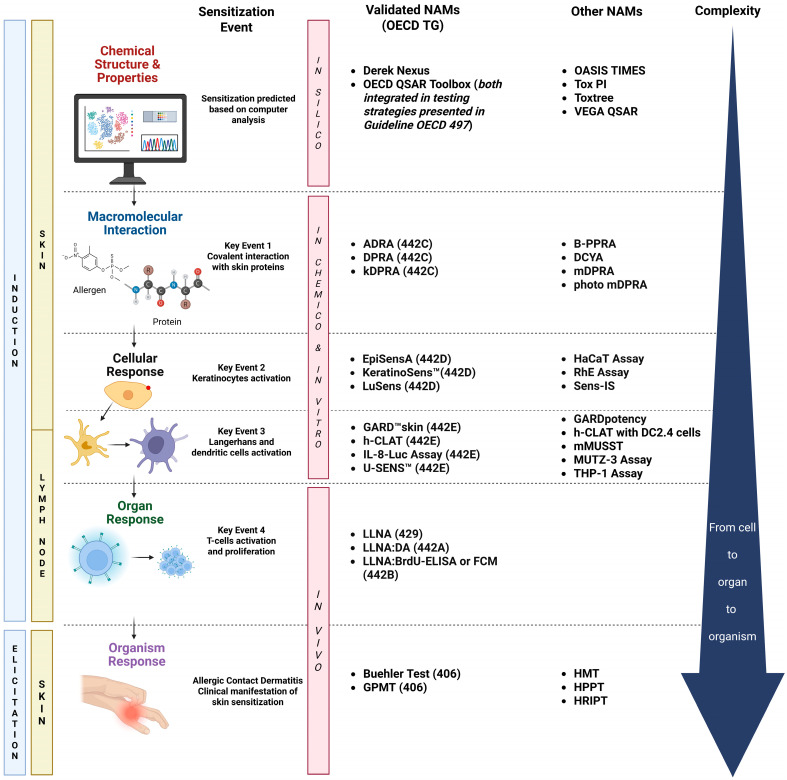
Overview of the skin sensitization Adverse Outcome Pathway (AOP) and New Approach Methodologies (NAMs) relevant to the assessment of substances inducing allergic contact dermatitis (ACD). The figure illustrates the four key biological events leading to skin sensitization, from molecular interaction to organism-level response, and highlights the corresponding validated NAMs (with associated OECD Test Guidelines) and additional emerging methods. Also shown are the in silico, in chemico, in vitro, and in vivo approaches currently applied or explored for evaluating sensitization potential across the complex product categories addressed in this review. ADRA, Amino Acid Derivative Reactivity Assay; B-PPRA, Peroxidase Peptide Reactivity Assay adapted for botanicals; BrdU, Bromodeoxyuridine; DA, defined approach; DCs, dendritic cells; DCYA, Dansyl Cysteamine; DPRA, Direct Peptide Reactivity Assay; ELISA, Enzyme-Linked Immunosorbent Assay; EpiSensA, Epidermal Sensitization Assay; FCM, Flow Cytometry Method; GARD™, Genomic Allergen Rapid Detection; GPMT, Guinea Pig Maximization Test; h-CLAT, Human Cell Line Activation Test; HMT, Human Maximization Test; HPPT, Human Predictive Patch Test; HRIPT, Human Repeat Insult Patch Test; IL, Interleukin; kDPRA, kinetic Direct Peptide Reactivity Assay; LLNA, Local Lymph Node Assay; mDPRA, modified Direct Peptide Reactivity Assay; mMUSST, modified Myeloid U937 Skin Sensitization Test; OECD, Organization for Economic Cooperation and Development; RhE, reconstructed human epidermis; QSAR, Quantitative Structure–Activity Relationship; TG, test guideline; TIMES, Times Metabolism Stimulator for Skin Sensitization; Tox PI, Toxicological Priority Index; VEGA, virtual models for property evaluation of chemicals within a global architecture. Created in BioRender. Costin, E. (2025) https://BioRender.com/3kjbofx (accessed 23 July 2025).

These tests are part of the wide umbrella of NAMs that cover the key steps of the Adverse Outcome Pathway (AOP) for skin sensitization (OECD Series on Testing and Assessment No. 168) [40]. Figure 1 describes the four key events (KEs) recognized as part of the AOP, which is based on a sequential chain of biological processes that follow the skin penetration by a sensitizer: (1) molecular interaction with skin proteins; (2) the activation of keratinocytes with the release of inflammatory mediators and induced gene expression associated with cytoprotective signaling pathways [e.g., antioxidant/electrophile response element (ARE)-dependent pathways]; (3) the activation of dendritic cells (DCs) associated with migration and antigen presentation in the lymph nodes; and (4) T-cell activation and proliferation [41]. The 4th KE is not currently addressed by OECD-validated NAMs and is therefore still evaluated using animal studies. The NAMs included in the AOP overlap with the induction and elicitation phases that take place in the skin and lymph nodes, amplify the allergic signal from macromolecular interactions with cells, and ultimately culminate in a whole-body response. The complexity of the NAMs also increases as the skin sensitization process progresses through the KEs, thus involving multiple cell lineages, which respond in physiologically specific ways (Figure 1).

Depending on their demonstrated capacity to predict a sensitizing KE, various NAMs have addressed the AOP as validated methodologies, while other emerging technologies have shown utility for the assessment of certain chemistries, as is detailed in further sections. The first KE (KE1) represents the covalent interaction between sensitizers with electrophilic profiles and the nucleophilic moiety in skin proteins. The in chemico validated NAMs such as the Direct Peptide Reactivity Assay (DPRA), the kinetic Direct Peptide Reactivity Assay (kDPRA), and the Amino Acid Derivative Reactivity Assay (ADRA) [42] are able to predict this molecular interaction [43]. The DPRA and ADRA are based on the use of High-Performance Liquid Chromatography (HPLC), while the kDPRA uses a fluorescence microplate reader to analyze adduct formation [44,45]. The relative simplicity and rapid nature of these in chemico assays make them straightforward platforms [46].

Cell-based alternatives are in vitro assays evaluating the antioxidant response element-NF-E2-related factor 2 (ARE-Nrf2) induction via a luciferase reporter, specifically the KeratinoSens™ and LuSens assays [47]. The KeratinoSens™ assay was validated first based on its capacity to address the KE2 represented by keratinocyte activation; it is one of the most widely used in vitro methods for assessing the skin sensitization potential of pure substances and formulations based on its reliability and effectiveness [46,48,49]. Another recently validated method to address the KE2 is the EpiSensA (Epidermal Sensitization Assay), an in vitro method that uses reconstructed human epidermis (RhE) to measure gene expression markers indicative of skin sensitization. This is the most human-relevant model among the NAMs discussed thus far, as it provides a complex, more physiologically meaningful platform compared to traditional cell lines [49].

The third key event (KE3) is assessed by the quantification of the expression of specific cell surface markers, genomic transcripts, chemokines, and cytokines, which characterize DC activation. Assays able to predict this KE include the Human Cell Line Activation test (h-CLAT), U937 cell line activation test (U-SENS™), Interleukin-8 Reporter Gene Assay (IL-8 Luc assay), and Genomic Allergen Rapid Detection (GARD™skin). The h-CLAT determines changes in the expression of cell surface markers in the human monocytic leukemia cell line THP-1 (i.e., CD86 and CD54), which are associated with the process of the activation of monocytes and DCs. U-SENS™ is a similar method, but it focuses solely on changes in CD86 expression in the human histiocytic lymphoma cell line U937 [50]. In contrast, the IL8-Luc assay evaluates shifts in IL-8 expression, a cytokine associated with the early activation of DCs in the THP-1-derived IL-8 reporter cell line (THP-G8, established from the THP-1 cell line) [51,52,53] using a luminescent endpoint. In 2023, the test method guideline was updated with a revised prediction model that enhances its applicability to poorly soluble chemicals based on the evaluation of cytotoxicity [54]. The GARD™skin provides a binary hazard identification of skin sensitizers [i.e., United Nations Globally Harmonized System (UN GHS) Category 1 vs. non-sensitizers]. The method evaluates transcriptional patterns of an endpoint-specific genomic biomarker signature, referred to as the GARD™skin Genomic Prediction Signature (GPS), in the SenzaCell™ cell line [51], a subclone of the myeloid leukemia cell line MUTZ-3 [50,52,53,55]. The NAMs included in the OECD 442 series have been described in previous publications [28,55,56]; therefore, detailed descriptions of each method are not in the scope of this review.

Based on the numerous validated NAMs capable of addressing the KEs within the AOP, several testing strategies have been established as DAs. For example, the two out of three (2o3) DA is intended for the identification of the skin sensitization hazard [i.e., UN GHS Category 1 vs. UN GHS Not Classified (NC)] and is not designed to provide information on the potency of a sensitizer or UN GHS subcategorization. In case the result of one of the test methods used in the 2o3 DA falls within the respective borderline range or is deemed inconclusive, a prediction can still be made if the outcomes of the other two test methods are concordant, conclusive, and have high confidence (i.e., results falling outside of the borderline range). The use and application of the 2o3 DA are described in the OECD guidance document 497 [20], which initially included defined borderline ranges for the DPRA, Keratinosens^TM^, and h-CLAT assays. In its most recent update, these ranges were also added for ADRA, LuSens, IL-8 Luc, U-SENS™, GARD™skin, and EpisensA. In some regulatory contexts, borderline results may still be considered conclusive if they meet the criteria of the assay’s prediction model. Still, results that are borderline require careful consideration, especially when using the 2o3 DA [57,58].

The Integrated Testing Strategy (ITS) DA, also outlined in OECD 497, uses quantitative data from the DPRA and the h-CLAT methods, along with an in silico prediction of skin sensitization provided by either Derek Nexus (ITSv1) or the OECD Quantitative Structure–Activity Relationship (QSAR) Toolbox (ITSv2). Both versions of the ITS can be used to predict skin sensitization hazard and potency subcategorization according to the UN GHS (subcategories 1A and 1B). In certain cases, a conclusive prediction may still be obtained from a single assay, provided that an in silico prediction within the domain of applicability is also available [20]. In the 2025 update of the OECD 497 guidance document, the respective ITS were revised to include ADRA as a source of KE1 information, while GARD™skin and LuSens were incorporated as inputs for KE3, thereby increasing the applicability of such DAs.

Recent advancements have enabled the development of NGRA strategies that use a derived point of departure (PoD) for skin sensitization based entirely on non-animal data. In this context, NAMs contribute hazard and potency information, which can be integrated with exposure estimates to conduct a full risk assessment. Among these approaches, a PoD in the form of a predicted LLNA EC3 values (pEC3) for use in risk assessment can be calculated for a chemical with input data from the kDPRA, KeratinoSens™ assay, and/or h-CLAT, along with the vapor pressure [59,60]. Several regression models are available to calculate a pEC3 value using partial data (kDPRA and KeratinoSens™ or h-CLAT; KeratinoSens™ and h-CLAT) or comprehensive data (all three tests). For recognized sensitizers, borderline results from individual assays may still be used, as the primary goal is to derive a PoD for relating the biological responses in test systems to human outcomes. Predicting an EC3 value offers the advantage of generating continuous potency values compared to predicting a chemical potency class. It also provides the opportunity to manage uncertainty using statistical tools based on knowledge of the accuracy of the prediction. Such uncertainty could be factored into refining the PoD value for conducting a skin sensitization risk assessment. If two of the cell-based tests are combined with the kDPRA, in addition to a binary outcome (hazard) using the 2o3 DA, the data can be used in the quantitative regression models to derive a PoD [59]. However, with this testing strategy and when used within a regulatory context, borderline ranges of the cell-based tests need to be taken into consideration for hazard identification [20,61]. It is, however, important to point out that while these methods provide data for hazard identification and potency estimation, a comprehensive risk assessment also requires evaluation of exposure levels under intended conditions of use.

A computational model developed to support quantitative risk assessment for skin sensitization using NAMs, and recently included in OECD 497 as a new DA, is the Skin Allergy Risk Assessment Integrated Chemical Environment (SARA ICE) [20]. This model integrates in chemico, in vitro, and, when available, animal and clinical data to estimate a human-relevant PoD in the form of an ED_01_, defined as the dermal dose expected to sensitize 1% of an HRIPT-eligible population [62]. In addition to the PoD, SARA ICE provides GHS classification probabilities, enabling simultaneous assessment of hazard and potency. Its flexible structure accommodates a variety of input data, offering a promising tool for regulatory applications that emphasize non-animal, human-relevant testing strategies.

Regardless of the approach considered, it is important to take into account the following aspects: (1) the chemical of interest is within the applicability domain of the selected test methods; (2) the input data used in the regression models are from valid experiments and are derived using the correct calculations described in the test method; and (3) the input data are of high quality.

Although these strategies are effective for individual substances, applying them to complex mixtures introduces new technical and regulatory challenges. It is important to distinguish between the technical applicability, meaning whether a material can be physically tested without interfering with the assay, and the predictive applicability domain, which refers to the chemical space for which a method or DA has been validated. While complex mixtures may be technically testable in assays covered by OECD 442 or OECD 497, these frameworks currently apply to defined substances. Results from mixtures may therefore fall outside of the validated scope and should be interpreted with caution. These considerations will be detailed in the analysis presented in the following sections.

## 3. The Challenging Landscape of BNP and UVCBS Within the Context of Skin Sensitization Assessment

### 3.1. Inclusion and Exclusion Criteria

This review focuses on both validated and non-validated NAMs applied to assess the skin sensitization potential of complex mixtures and formulations. Through the review of available sources, 37 manuscripts were identified that report on the evaluation of skin sensitization for the categories of interest and cross-referenced with NAM data. The search and identification of these manuscripts was conducted using primarily the PubMed database and the individual websites of the journals the papers were published in.

The search terms used to narrow the resources were separated into two categories: pertaining to the NAMs and relevant to the product classes of interest, respectively. The terms referring to the NAMs that were used for our analysis were “in vitro”, “NAMs”, “in vivo”, “clinical”, “ex vivo”, “skin sensitization”, “sensitizers”, “regulatory”, “validated”, “non-validated”, “QSAR”, “ADRA”, “DPRA”, “kDPRA”, “EpiSensA”, “KeratinoSens™”, “LuSens”, “GARD™skin”, “h-CLAT”, “IL-8-Luc Assay”, “U-SENS™”, “LLNA”, and “GPMT”. The terms indicating the non-validated NAMs listed in Figure 1 were not specifically searched for; they were, however, associated with the validated NAMs in the manuscripts identified and also with the product classes of interest. The search terms used for the product classes were “mixtures”, “multi-constituent”, “extracts”, “botanicals”, “BNS”, “UVCBs”, “medical device”, “wearables”, “agrochemicals”, “pollutants”, “finished formulations”, and “final products”.

As for the inclusion criteria, the manuscripts considered for the analysis had to associate the product classes (of choice, see also the exclusion criteria) and the NAMs capable of addressing their skin sensitization potential. Also considered were the manuscripts reporting on both NAMs validated and those not validated for regulatory purposes. The manuscripts that met the following exclusion criteria were not considered for our analysis: non peer-reviewed; investigating product classes other than those of interest to our analysis; and even though they are of high interest and considered complex mixtures and finished products, cosmetics and personal care products were not included in the review as these classes are extremely wide and have been addressed in publications dedicated specifically to their topic [63,64,65,66,67]. Also excluded from our analysis were other categories such as impurities, nanomaterials, microplastics, etc. Our analysis identified a total of 11 manuscripts relevant to the botanicals: 9 for mixtures, 8 for medical devices/wearables, 7 for agrochemicals, and 2 for pollutants.

### 3.2. Complex Mixtures Used as Tools for Evaluation of Skin Sensitization NAMs

Among approaches to address human health risk assessment of mixtures, one possibility is based on deriving the combined toxicity of individual components primarily from data generated on single chemical constituents. A more precise approach is to test complex mixtures and finished products, generating results that reflect the exposure to the entire mix of ingredients all at once. Furthermore, several DAs were published to predict the skin sensitization hazard [20], but they were based mainly on the testing of simple and “easy-to-test” substances. In this context, strategies addressing “difficult-to-test” mixtures and finished products need to be developed.

Although all product classes identified in our analysis involve mixtures or generally “difficult-to-test” substances, a total of nine manuscripts focused on complex mixtures were selected (Table S1). Their reported safety assessment in terms of skin sensitization was conducted using a wide range of assays, covering the entire spectrum of NAMs. Furthermore, the mixtures were intended to mimic a diversity of products, from cosmetic/personal care to plasticizer product lines.

Validated NAMs were primarily considered as they were demonstrated to address several KEs of the AOP, such as DPRA, h-CLAT, and KeratinoSens™ assays. In certain reports, some combinations of these assays were used [68,69,70]; however, it is unclear if these assays were selected based on a particular DA or on the expertise of the laboratory conducting the testing. Several NAMs not validated for regulatory purposes or in silico tools were also used [71,72,73].

Most of the mixtures were used to evaluate the NAMs’ performance in identifying ingredients reasonably known to act on the sensitization KEs specific to the assays used. These ingredients were selected at concentrations relevant to common use products [39,68,73,74]. These mixtures were based on ingredients that were easier to test than finished products, at the very least from the solubility perspective, which is an inherent challenge, especially for cell-based assays. For example, aldehydes with various skin sensitization potentials were mixed and tested using the DPRA. The experiments revealed certain shortcomings or artifacts encountered when using this method for aldehyde assessment [39,68,74], especially with regard to the interaction of the mixtures with the peptides used in the assay or to the concentrations tested (Table S1). The assay was capable of identifying sensitizers within a mixture when their potential was relatively different (a strong sensitizer paired with a weak one). In general, in these experiments, the sensitization potential of each ingredient was known from historical animal data (primarily LLNA) or from human studies. In one study, the sensitizers were mixed with known skin irritants, and the synergistic effects were evaluated using the KeratinoSens™ assay, which proved to be a valuable tool for the quantitative detection of sensitizers within mixtures [75].

The importance of the base/carrier composition was thoroughly investigated in experiments using the Sens-IS assay, which is a NAM awaiting completion of the validation process [71]. Like in previous examples, sensitizers with various skin sensitization potentials were used at various concentrations and prepared in five different base types to allow for addressing the importance of the vehicle for the bioavailability through a reconstructed tissue model. Thus far, this is the only manuscript reporting on the importance of the base for this endpoint that we identified in our analysis.

Complex products of unique design, such as plasticizers (based on a mixture of isomers) and UVCBs derived from the petrochemical industry, have been evaluated for their skin sensitization potential using NAMs [69,72]. Given the complexity of such products, several NAMs were used in addition to in silico tools in an integrated Weight of Evidence (WoE) approach, where all the available information was used for the final skin sensitization assessment.

The precise selection of the mixtures included in Table S1 helped the identification of some common challenges encountered when NAMs are used to address skin sensitization and opened the possibilities to analyze even more complex mixtures and finished products, as detailed in the sections that follow.

### 3.3. Botanical Extracts

Botanical mixtures as active ingredients are of a continuously increasing interest, especially to a widespread assortment of cosmetic and personal care products, including aromatherapy, detergents, household, and perfume products [14]. They also hold great potential for the development of new drugs, agrochemicals, and dietary supplements [15,76,77,78,79]. More than 70% of the US population has been reported to use a herbal product, often based on consumers’ desire for alternative natural products, with additional benefits over “classical” (i.e., chemical) ingredients [80]. It has been reported that in the four-year period from 2015 to 2019, an annual 10–11% growth occurred in the global “natural” cosmetics market [81]. The anti-aging cosmetics sector has registered a considerable use of botanical ingredients, with over 70% of the products on the market containing at least one botanical source ingredient [82].

The marketplace has indicated that the consumer preference for products that contain BNS is based on a perception that “natural” is healthier, organic, ecological, and, by default, safe [83]. Contrary to popular beliefs, natural products are not necessarily free of toxicity. Thus, risk evaluation of the raw materials and finished products must be carried out to meet regulatory criteria and obtain information related to human safety [14]. Over the past few years, proposals for the safety assessment of BNSs have been made, in particular for systemic toxicity endpoints [84] with the main focus on the use of BNSs as dietary or food supplements [9,85]. However, topically applied products containing BNSs are often not evaluated for their efficacy (e.g., therapeutic or cosmetic) or risk of undesired effects, the most common being irritation or ACD [80]. Therefore, an evaluation of BNSs for skin sensitization potential must be a key part of the overall safety assessment process [86].

The stability and optimal use concentrations of botanicals are also unregulated, therefore indicating a critical need to characterize botanicals and to determine a concentration that is safe for human use. One of the primary challenges encountered when testing the safety of botanicals, and particularly the skin sensitization endpoint, is related to their physico-chemical properties. They are supplied in a wide variety of preparations, ranging from highly concentrated powdered plant materials and solvent-based extracts to distilled essential oils, expressed juices, tinctures, waxes, vegetable oils, lipids, or plant purified components. Even within the same plant species, the composition can vary based on growth location, soil and weather conditions, and harvest time [86,87]. Adding to this complexity, there is limited experience in performing risk assessments on complex mixtures and finished products using animal tests. There is even less experience on whether the novel in vitro tests could be applied to inform on hazard and risk. Furthermore, the skin sensitization testing for those materials intended for use in cosmetics marketed in the European Union must be accomplished without the use of animals in the context of the current regulatory requirements [10].

Although the use and acceptance of NAMs have increased in the past decade, there are no established testing strategies applicable to botanicals. Indeed, they are considered outside the applicability domain of most OECD-adopted NAMs due to their complexity and compositional variability [86]. In support of the efforts to use NAMs for the assessment of skin sensitization and of other critical endpoints, the Research Institute for Fragrance Materials (RIFM) published its approach to evaluate natural complex substances (NCS) [88]. A decision tree was proposed, starting with an evaluation of all available data. When insufficient data are available, the whole NCS can be evaluated using the reactive Dermal Sensitization Threshold (DST) of 64 μg/cm^2^ [89,90,91]. If dermal exposure of the NCS is above the reactive DST, component-based assessment and further data generation could be conducted. Recently, a framework following a tiered approach was published [36,92], which outlines an integrated WoE strategy for sensitization risk assessment. This NGRA approach involves reviewing existing information, calculating a NAMs-based PoD, assessing uncertainty, and relating findings to consumer exposure. This proposed framework could also be reapplied for BNSs.

A total of 11 manuscripts investigating the skin sensitization potential of botanicals, which were published between 2013 and 2024, were identified (Table S2). This category yielded the highest number of studies, which is unsurprising given the widespread use and availability of botanical products and associated data sets. Most of the plants investigated in these manuscripts were unique. The majority of the paired animal data were generated in the LLNA and GPMT, while the human data were obtained in HMT and HRIPT studies, and no relevant studies addressing accidental exposure were identified. The analysis indicated a preference for using in silico tools such as Derek Nexus and OASIS Tissue Metabolism Simulator (TIMES) to characterize the sensitization profile of the botanical constituents. However, the use of in silico tools proved to be challenging given that most of the extracts are not characterized and their component concentrations are rarely provided or precise. Among the NAMs evaluated, the explored validated assays were: DPRA, KeratinoSens™, h-CLAT, U-SENS™, and LuSens, either as single assays [14,93], or in various combinations that allowed interpretations based on the 2o3 DA [17,94,95]. The Sens-IS assay was the only method among those evaluated that utilized a reconstructed skin model [17,95], based on its capacity to provide information on the potency of skin sensitizers [95]. Alongside Sens-IS, some other NAMs that are not yet validated for regulatory purposes were considered either as stand-alone or in various combinations with established NAMs [17,18,86]. Some may have been modifications of validated assays such as the micro DPRA (mDPRA) [15] and the h-CLAT adaptation using DC2.4 cells [4]. These adaptations simplify the significance of the experimental work, given that a substantial part of the method has already been evaluated.

The properties of botanicals allowed for spiking experiments to evaluate new assays like the Dansyl Cysteamine Assay (DCYA) [18] or the Botanicals Peroxidase Peptide Reactivity Assay (B-PPRA) [86]. Spiking experiments were also used as surrogate approaches to investigate the validated assays for their capability to identify low concentrations of skin sensitizers, if present in the botanical extracts [14].

Several challenges were identified regarding NAMs’ capability to address the skin sensitization of botanicals. For instance, the lack of proper characterization of the botanical products hinders meaningful comparison of their skin sensitization potential with existing reference data [55,63]. Regarding the applicability domain of cell-based assays, significant limitations were identified for their use with oil-based botanical extracts [94]. Tannins were reported as a challenging chemical class since they are known to form complexes with proteins via hydrogen bonds and hydrophobic interactions, which might affect the KE1 methods, such as the mDPRA [15]. Similarly, in chemico methods may fail to detect botanical sensitizers that require metabolic or oxidative activation, as in the case of pre- or pro-haptens, highlighting the application for modified assays like the B-PPRA that incorporate an oxidative step using horseradish peroxidase and hydrogen peroxide [86].

These findings highlight that the complexity and compositional variability of botanical extracts require sophisticated strategies to determine their skin sensitization potential. As such, the accuracy of the 2o3 DA using various combinations of three validated assays (DPRA, h-CLAT, and LuSens) and also in combination with two other non-validated NAMs [modified Myeloid U937 Skin Sensitization Test (mMUSST) and Sens-IS] [17] was evaluated. The authors concluded this strategy is not recommended for botanical extracts and that some substance subgroups may not be within the applicability domain of the methods used. A complex WoE approach that integrated human clinical and animal data alongside data generated in validated (DPRA, h-CLAT, and KeratinoSens™) and non-validated NAMs (Sens-IS) has been proposed [95]. In their experiments, the authors established a unique set of 14 botanicals that can be considered as a reference set for sensitization potential and potency, supporting both the evaluation of existing methods and the development of new DAs for this endpoint.

### 3.4. Medical and Wearable Devices

This section addresses two categories of complex products: medical and wearable devices. These were selected due to their unique properties, intended use, and the fact that they are typically evaluated as extracts, placing them within the category of complex mixtures. Medical devices fall under the jurisdiction of the US FDA or equivalent regulatory agencies in other countries, while wearables are not regulated, per se, but they are evaluated thoroughly by their manufacturers in order to avoid human adverse effects. In terms of definition, according to the US FDA, a medical device is “any instrument, machine, contrivance, implant, in vitro reagent that is intended to treat, cure, prevent, mitigate, or diagnose disease in man” [96]. The medical devices could be implantable or not; therefore, their biocompatibility evaluation is conducted to address exposure to skin and the specific target organ.

Wearables, on the other hand, cover a wide spectrum of products, ranging from multifunction watches, activity bracelets, smart glasses, next-generation earphones, and rings with integrated technology to smart clothes or safety goggles. All these products are of a very complex design and contain parts manufactured using drastically different chemical components, such as batteries, monitors, wires, cameras, sensors, screens, etc. Besides a polymer base, they also contain adhesives, plasticizers, catalysts, initiators, cross-linking agents, processing aids, UV stabilizers, pigments, and antimicrobial agents [97,98]. When compared to medical devices, the wearables are similar in that the materials they are made of are in direct contact with the consumer’s skin for extended periods of time, and thus pose a similar threat with regard to safety. These products have been associated with ACD through leachable ingredients and impurities that might be extracted and penetrate the skin [99,100].

Biocompatibility testing is a key element within the development and regulatory approval of medical devices by ensuring their safety when exposed to humans. For this goal, the International Organization for Standardization (ISO) developed a set of standards referred to as the ISO 10993 series. These guidelines provide a framework for evaluating the biocompatibility of medical devices through a risk-based approach, guiding the selection and application of biological tests to ensure patient safety. Extractable and leachable studies are common experiments used to evaluate medical devices for consumer safety. Testing protocols for medical devices have been used as guidance in assessing the skin sensitization potential of wearable devices, often with modifications to better reflect real-life exposure scenarios.

One key aspect of the ISO methodology for biocompatibility testing is the requirement to evaluate the skin sensitization risk, described in ISO 10993-10 [101]. Currently, the standards recommend using in vitro test systems as tools for screening purposes prior to animal testing [101]. The fourth, and current, edition of ISO 10993-10 encourages the use of in vitro data in a step-wise fashion within an Integrated Approach to Testing and Assessment (IATA); however, it acknowledges that the applicability of in vitro and in chemico approaches for testing medical devices is not yet established [101]. Without substantial data supporting the adaptation of in vitro and in chemico test systems for testing medical devices, there remains reliance on in vivo assays such as the GPMT, Buehler test, and LLNA.

The specific preparation of samples and reference materials is described in ISO 10993-12 [102]. It is often the case that medical devices will need to undergo an extraction procedure in order to prepare a solution that can be used for exposure to the test system. Notably, extracts must be prepared in both polar (e.g., water, saline, culture media without serum) and non-polar (e.g., freshly refined vegetable oil, cottonseed or sesame oil) vehicles. Alternative solvents are permitted with scientific justification, such as ethanol/water, ethanol saline, polyethylene glycol 400 (diluted to a physiological osmotic pressure), Dimethyl sulfoxide (DMSO), and culture media with serum [102].

A total of eight manuscripts, three of which analyzed the skin sensitization of wearables [7,103,104] and five of which investigated medical devices [105,106,107,108,109], were included in this literature analysis (Table S3). For most of the cases, validated assays have been considered (DPRA, KeratinoSens™, h-CLAT, LuSens, GARD™skin, and kDPRA) either alone or in combinations that allowed the use of the 2o3 DA or to derive a pEC3 value based on the evaluation of device constituents [104]. The preponderance of the validated assays and the lack of in silico tool usage might be derived from the fact that medical devices are strictly regulated and there is little to no flexibility in using methods that have not reached the phase of validated standards or that are currently not applicable to complex mixtures. The choice of assays might also be related to the proficiency of the various laboratories performing the testing. Like in the case of botanical extracts, experiments using known skin sensitizers spiked into the device extracts were used [105,106,108,109] to evaluate the sensitivity and predictive power of the NAMs for the skin sensitization endpoint.

Besides assay-specific challenges identified (Table S3), several general technical difficulties were found with regard to the medical devices, such as their degradation, low concentrations of extractable toxicants in the composition, and challenges in sample preparation [6]. These challenges show the need to employ complex strategies for testing and data interpretation. In particular, several of the testing strategies included endpoints from multiple KEs of the skin sensitization AOP [103,104,107,108] (Figure 1) that subsequently enabled data interpretation via the 2o3 DA. In addition to increasing confidence in predictions, another inherent benefit of testing multiple KE endpoints is the ability to mitigate the limitations posed by each individual assay. For instance, a limitation of the DPRA is the inability to test metals and inorganic compounds due to their reactions with proteins via mechanisms other than covalent binding [42].

Several emerging NAMs are currently being qualified as test systems for use in the testing of device extracts. These newer generation assays represent KE2 (Sens-IS & EpiSensA) and KE3 (GARD™skin). Both EpiSensA and GARD™skin have been adopted by the OECD. These newer assays all analyze changes at the genomic level in response to skin sensitizers, which provides mechanistic insights that are not captured by other previously validated assays. The EpiSensA was performed in concert with other NAMs to identify the sensitization potential of a known sensitizer, 2,4-dinitrochlorobenzene (DNCB), which was spiked into extracts of polyurethane [108]. While each of the assays was ultimately able to correctly predict the sensitizing agent, it is notable that only the non-polar extraction was predicted correctly in the EpiSensA. The Sens-IS assay showed high predictive accuracy by correctly identifying the vast majority of tested sensitizers and all non-sensitizers in extracts of medical devices [109]. Notably, the two misclassified sensitizers were associated with poor solubility in the extraction solvents, and expected classification was achieved when tested at higher concentrations, suggesting that solubility limitations may influence assay outcomes.

Adaptations or optimizations of established assays are facilitated by the fact that most of the technical aspects have already been validated. For example, the GARD™skin was adapted to polar and non-polar extracts, including olive oil and sesame oil, which allowed the correct prediction of the sensitization potential of medical devices [105].

Some key differences in extraction methods include the fact that medical devices are extracted under exaggerated conditions (to address the worst-case scenario), while wearable devices are generally tested under intended-use conditions. For example, the extraction conditions used to prepare DNCB-spiked polyurethane extracts did not follow the ISO 10993-12 standards [108]. When ADRA was used, the device was extracted in both water and acetonitrile, and at a lower temperature than specified in ISO 10993-12:2021 [102] (Table S3). While water is an acceptable polar extraction vehicle, acetonitrile is not listed as a recommended extraction vehicle. In the h-CLAT testing of DNCB-polyurethane extracts, complete culture media was the only extraction vehicle used. While this does not satisfy the condition of extracting the medical device in both a polar and non-polar vehicle, ISO 10993-12:2021 [102] does allow for alternative extraction vehicles, such as a culture medium with serum, with justification. It is likely the case that a complete culture medium was selected as an appropriate extraction vehicle to compensate for the h-CLAT’s limitation of testing hydrophobic substances, such as an extract prepared in a non-polar extraction vehicle [50,108,110]. The novelty of this study lies in the qualification of DNCB-spiked polyurethane sheets as effective extractable positive reference materials that closely resemble the product lines being tested. Since identifying a “designer” polymer as a skin sensitizer is difficult, spiking experiments are essential to establish suitable reference materials for use in medical device testing. Some other studies that aimed to adapt testing conditions to better reflect real-world exposure used neutral artificial sweat to extract various wearable devices, simulating practical use conditions [7]. This approach improved the relevance of h-CLAT assay results, supporting its utility in identifying potential allergens in everyday items like watch bands and emerging personal care products.

Another type of spiking experiment was conducted using the Sens-IS assay to identify the sensitization potential of extracts from medical-grade silicone impregnated with various well-characterized sensitizers [106]. Ultimately, the assay was able to correctly identify five of the six sensitizing agents in the medical device extracts (four materials were categorized as GHS 1A, one material categorized as GHS 1B). The incorrectly predicted weak sensitizer, phenyl benzoate (category 1B), was later correctly predicted when exposed to exaggerated extraction conditions.

The 2o3 DA was used to evaluate the results of extracted wearables [103], showing how mechanistically complementary NAMs enhance hazard identification for complex mixtures. A different approach focused on evaluating constituents of concern commonly used in wearable devices used in vitro data from the kDPRA and KeratinoSens™ assay within established regression models [59,60] to derive a predicted LLNA EC3 (pEC3) for each compound. The results correlated with previous reports of ACD induced by such constituents in humans. The use of tools such as pEC3 expands the abilities of the OECD-adopted in vitro and in chemico assays to conduct quantitative risk assessment [104].

There is growing interest in using NAMs for skin sensitization assessment of medical devices. Similarly, the evaluation of wearable devices relies on the methods applied to medical devices, given their similar conformation, and is based on learnings applicable to multiple industries such as personal consumer products and cosmetics. Continued development in this field depends on the successful validation and acceptance of NAMs.

### 3.5. Agrochemicals

Skin sensitization induced by agrochemicals, whether from active ingredients or formulations, is a concern for both agricultural workers and consumers. Therefore, assessing the sensitization potential of this product class is crucial to protect health and meet regulatory requirements.

A total of seven manuscripts were identified in this category (Table S4), focused primarily on glyphosate-containing formulations, POEA-based formulations, silicone-based compounds, methacrylate esters, and surfactant mixtures. These are typically multi-constituent mixtures, where each component may influence the overall sensitization response.

Validated NAMs for regulatory purposes were primarily used in the manuscripts analyzed, either individually [111] or in combinations supporting the 2o3 DA or WoE approaches [5,46,48,112,113]. Of the cell-based assays, the KeratinoSens™ assay is one of the most widely used in vitro methods for assessing skin sensitization potential of agrochemicals with high accuracy and in alignment with in vivo data. This assay is well-regarded for its reliability [46,49] and can be used for testing both pure substances and formulations [48,49]. However, solubility issues and undefined molecular weights often require adaptation of the KeratinoSens™ protocol [49]. Indeed, the KeratinoSens™ method was adapted to assume a common molecular weight and to consider formulations as single entities rather than individual components [111]. Through these implementations, the assay correctly predicted three out of four sensitizing formulations and all six non-sensitizing formulations when compared with in vivo data.

Another commonly used assay for assessing agrochemicals is the DPRA. Its simplicity and rapid nature make this assay not only relatively easy to perform [46] but also amenable to modifications that improve its predictive capacity. As such, mDPRA (10-fold reduction in the reaction volume of the DPRA) and photo mDPRA (further modified by adding an irradiation step) were developed to test glyphosate and POEA-based agrochemicals. In these studies, mDPRA correctly predicted Glyphosate AKB 80 and Roundup Transorb R as sensitizers, in agreement with human data. Similarly, photo mDPRA predicted most sensitizers correctly [112]. A common challenge with DPRA is that it may not accurately predict all types of sensitizers, particularly those requiring metabolic activation. Moreover, DRPA only assesses the initial peptide binding step and does not specifically include an S9 mix or other external metabolically active factors. These challenges are, however, easily addressable by incorporating complementary testing, such as the KeratinoSens™ or h-CLAT assays, which can cover a broader range of sensitization mechanisms [46] and thus allow a more complete evaluation by the use of the 2o3 DA. Furthermore, in vitro models that incorporate metabolic activation can complement DPRA by helping identify a wider range of sensitizers.

EpiSensA offers a more physiologically relevant model than traditional cell lines. Within a panel of 20 tested agrochemical formulations, EpiSensA predicted 16 correctly. Based on this data set, the EpiSensA was considered a reliable predictor for methacrylates and silicone-based compounds [113]. This assay is inherently complex, requiring specialized equipment and expertise for gene expression analysis. Moreover, the solubility of chemicals in the vehicle control and variability in the reconstructed human epidermis models can impact reproducibility across batches. However, collaborative efforts to develop standardized protocols for RhE-based models can improve the consistency and reproducibility of results [49].

GARD™skin, recently included in OECD TG 442E, was evaluated using 42 agrochemical formulations and demonstrated 76.2% overall accuracy, with 85.0% sensitivity and 68.2% specificity compared to historical animal data [114]. GARD™potency, its non-validated counterpart for potency assessment, correctly subcategorized 14 of 17 sensitizers by GHS category. Together, these results support the applicability of these assays to agrochemical formulations and highlight the value of integrated approaches. Across the studies reviewed, the aforementioned assays have shown significant success in predicting the skin sensitization potential of agrochemicals. Their combined use in integrated testing strategies provides a holistic assessment of chemical safety, enhancing overall predictive power. For agrochemical formulations, the 2o3 DA demonstrated the highest balanced accuracy (78%) for predicting in vivo skin sensitization hazards, making it the most reliable strategy among those evaluated for this complex product class [5]. The primary challenges faced by these assays include solubility of the chemicals and vehicle controls and, hence, their bioavailability to the test systems, the variability of cell-based assays, and the need for standardized protocols for complex formulations. Assessing complex mixtures and finished formulations can be challenging due to potential synergistic or antagonistic effects. Addressing this challenge requires an IATA that allows for combining data from multiple assays to provide a comprehensive evaluation [115,116].

## 4. NAMs and Associated Challenges

The applicability domain (AD) refers to the range of physicochemical, structural, or biological space for which a test method or model is expected to provide reliable predictions. For this manuscript, that endpoint is skin sensitization. If a chemical falls outside the AD, confidence in the prediction decreases, limiting the method’s applicability. The AD of an assay is used in part to define its limitations as discussed in its respective test guideline and in various publications summarized herein. The validation studies are not usually conducted to cover the entire chemical class or product type universe. The performance of the validated method relies upon the experience gained after the guidelines are adopted and used for the testing of candidates designed by various industry branches.

**Figure 2:**
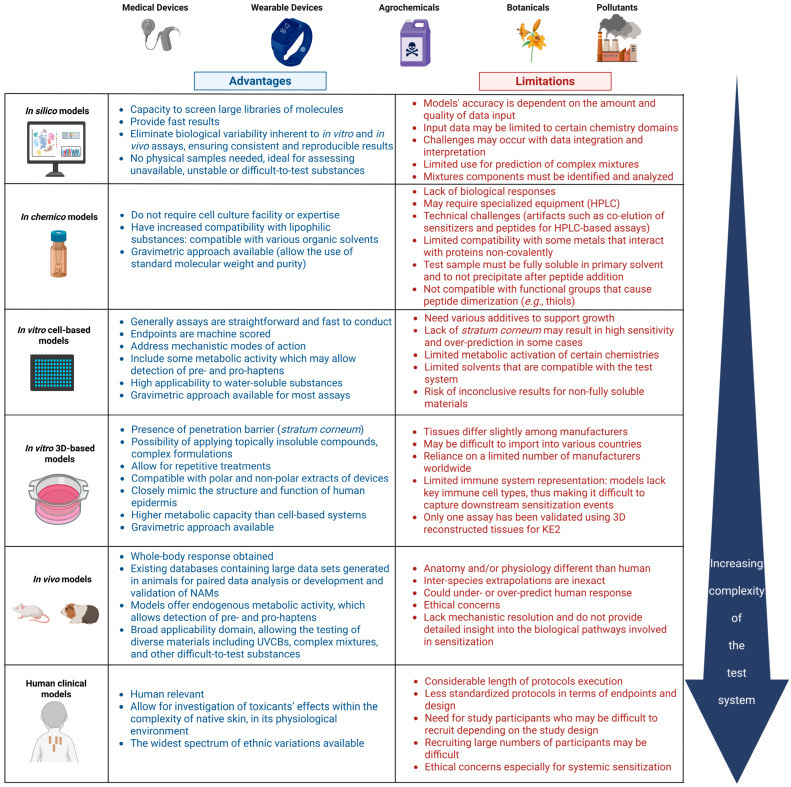
Summary of the advantages and limitations of NAMs for skin sensitization of complex mixtures, arranged by increasing biological complexity, from in silico to clinical models. Abbreviations: 3D, three-dimensional (referring to reconstructed tissue models); HPLC, High-Performance Liquid Chromatography; KE2, Key Event 2; UVCBs, Unknown or Variable Composition, Complex Reaction Products or Biological Materials. Created in BioRender. Costin, E. (2025) https://BioRender.com/sz4xafn (accessed 23 July 2025).

Most stand-alone methods have their own specific limitations that can be overcome when combined to allow for a prediction to be made [28]. These limitations include unsatisfactory accuracy and predictive capacity, especially when the chemistries investigated are outside the AD. The single method might not provide all possible outcomes of interest or properly address the mechanisms of action alongside Absorption–Distribution–Metabolism–Excretion (ADME); they might represent a single KE in the AOP or of the complex in vivo process. Potency prediction in the case of skin sensitization might be a limitation of the assays; data should be placed in the wider context of integrated data and evidence from different studies. The general and specific advantages and limitations of the NAMs used to assess the skin sensitization potential of complex mixtures and finished products, presented from the perspective of the test systems’ complexity, are summarized in Figure 2. This section provides a detailed discussion of the specific challenges associated with testing certain chemistries using the NAMs identified in our analysis as applicable to complex mixtures and final formulations.

While it is conceivable that pre- and pro-haptens may be outside of the AD of the current OECD NAMs for skin sensitization, many such chemicals have been correctly identified by these assays. For example, the DPRA and kDPRA were not designed to detect the sensitizing properties of pro- and pre-haptens. Nevertheless, both have in some cases correctly detected peptide reactivity [117,118,119]. In contrast, KeratinoSens™ and h-CLAT may yield negative results for pro- and pre-haptens due to limited metabolic capacity [120]. Positive results may still be valid, but negative outcomes should be interpreted with caution. In silico tools such as the OECD Toolbox and Oasis TIMES-SS can assist in identifying pre- and pro-haptens. This challenge is also seen when testing botanicals or natural extracts, which often contain unstable or unknown components that may require metabolic activation [16,49]. Modified approaches incorporating oxidative conditions (e.g., horseradish peroxidase–hydrogen peroxide) have shown potential to enhance peptide reactivity detection for substances requiring activation, including pre- and pro-haptens [85]. These findings underscore the need for integrated strategies combining NAMs with chemical profiling, especially for materials requiring metabolic activation.

Solubility of the test chemical, or device extract, in assay-appropriate solvents can be a limitation for all NAMs considered for the skin sensitization endpoint. For example, if a chemical cannot be dissolved in an appropriate solvent to obtain a final concentration of 100 mM for the DPRA, it may still be tested at a lower concentration. In this case, a positive result can be used to support classification as a sensitizer, but a negative result is considered to be inconclusive. For the kDPRA, if a test substance cannot be dissolved in phosphate buffer or acetonitrile, a log k_max_ > −2.0 may still be used to identify the chemical as a UN GHS 1A sensitizer. However, the assay prediction model may not be used for predicting a UN GHS 1B sensitizer/non-sensitizer result [42]. In the case of using the kDPRA for the derivation of a pEC3, a test substance may be tested at lower soluble concentrations and the log K_max_ may still be applicable for use in such calculations [104]. For the cell-based methods that generally use an aqueous culture medium, highly lipophilic chemicals may be difficult to test due to solubility challenges posed by their properties. Therefore, it should first be determined if the test substance dissolves in the exposure medium or at least forms a stable dispersion (e.g., a colloid or suspension that does not separate from into different phases). This consideration is especially relevant when evaluating medical or wearable devices, as polar extracts (e.g., oils) may not be compatible with aqueous cell-based systems. Despite these limitations, newer assays like Sens-IS, EpiSensA, and GARD™skin have shown strong performance, using gene expression as a readout to detect sensitizers in polar extracts [105,108,109]. These assays share a common readout based on the gene expression profiles of specific marker genes, suggesting that gene expression analysis may offer greater sensitivity for detecting low-concentration sensitizers present in device extracts. In the context of medical devices, additional efforts have been made to adapt established sensitization assays to identify leachable ingredients with sensitization potential, for example, by using culture media as an extraction solvent to enhance extract compatibility with cell-based assay systems [103].

Complex mixtures and finished product extracts represent a significant challenge for all NAMs given that their composition may be unknown. Originally, the prediction model for the DPRA could not be used for UVCBs due to the need for a defined molar ratio of test substance and peptides; however, some essential oils have been tested using a gravimetric approach and are reported to be positive in the DPRA [55]. They were also confirmed to induce KEs 2 and 3, as shown by the KeratinoSens™ and h-CLAT assays, respectively. This gravimetric approach has been expanded to multi-constituent mixtures of unknown composition, which can now be tested in the DPRA using a single purity and apparent molecular weight [42]. In one study assessing botanicals, an apparent molecular weight was estimated based on chemical characterization of essential oils, allowing peptide depletion measurements to be expressed in molar terms and supporting DPRA testing of complex mixtures [93]. Similarly, agrochemical formulations have been tested in the DPRA using a fixed molecular weight value; this approach allows the application of the assay when the exact composition is not fully defined or when component-specific concentrations cannot be determined [5]. In the context of medical device safety assessment, an adapted DPRA approach was used in which defined volumes of device extracts were directly tested without calculating molar concentrations or requiring molecular weight assumptions [107]. This strategy further reflects the practical need to evaluate extracts from materials such as latex, silicone, and essential oil-containing products, where exact composition and sensitizer concentration are often unknown. However, the occurrence of three false negative outcomes among 42 tested samples highlights a limitation of this approach, particularly when dealing with complex or poorly characterized materials where sensitizing components such as residual impurities, processing additives, or synthetic fragrances may not be adequately captured by the in vitro assays. Similarly, a recent report that used gravimetric procedures with assumed default molecular weights of 375 or 500 g/mol to evaluate plant extracts and polymeric materials highlighted ongoing concerns about the inconsistent handling of test substances with undefined molecular weights in OECD-adopted skin sensitization NAMs [17,121,122]. Currently, there is no standardized default molecular weight across these assays, and the choice of value can significantly influence test results. To reduce variability, empirically derived default molecular weights supported by experimental data may provide a more consistent basis for assay interpretation.

When testing these challenging product categories, particularly for in vitro systems, consideration should be given to possible interference of cytotoxic constituents with the observed responses. For example, in the KeratinoSens™, the presence of high content non-sensitizing cytotoxic constituents may mask the response of weakly sensitizing or sensitizing components present at low concentration [48]. This is because, regardless of the level of gene induction, a cytotoxic test sample may reduce cell viability to the extent that the result appears as a false negative. This must be evaluated case-by-case, as some reports indicate that allergens combined with irritants lower the sensitization threshold [75].

Some other considerations are to be taken into account for validated NAMs. Metals and inorganic compounds known to react with proteins by mechanisms other than covalent binding should not be tested in either the DPRA or the kDPRA. However, some metal salts (e.g., cobalt chloride and nickel chloride) showed reactivity in the DPRA, indicating activation of KE1 in the AOP for skin sensitization [123]. Similarly, it has been shown that the DPRA can correctly identify the sensitization potential of platinum compounds, including recognized respiratory sensitizers [124] (which are outside of the scope of this paper). Indeed, such limitations are especially critical for metal-containing devices; for example, nickel-containing orthopedic implants have been shown to provoke type IV hypersensitivity reactions, such as ACD, which present as localized inflammation near the implant site. These reactions have been confirmed through clinical follow-up and supported by patch testing in multiple reported cases [125].

Furthermore, test substances that do not covalently bind to cysteine but promote its oxidation (i.e., cysteine dimerization) may lead to an overestimation of cysteine depletion in both the DPRA and kDPRA and may potentially lead to a false positive prediction or assignment to a higher reactivity classification (e.g., DMSO and some aldehydes such as farnasal) [126]. For the DPRA, results obtained with test chemicals that absorb significantly at 220 nm and have the same retention time as the peptide (e.g., co-elution) may be difficult to interpret. Data for chemicals that co-elute with both peptides or just the cysteine peptide are considered to be inconclusive. When co-elution occurs with only the lysine peptide, the “cysteine-only” prediction model can be used.

The kDPRA is not appropriate for chemicals that react exclusively with the lysine peptide since the assay uses only a cysteine-containing peptide (e.g., some acyl-halides, phenol-esters, or aldehydes) [119]. Test chemicals that contain a primary thiol group or release a thiol group upon decomposition will react with the detection probe and therefore cannot be assessed in the kDPRA. Likewise, fluorescent chemicals with excitation in the range of the detection probe–peptide adduct (390 nm) or chemicals that absorb in the emission range of the detection probe–peptide adduct (480 nm) can cause interference with the assay. Guidance on evaluating these situations is provided in the DB-ALM Protocol no. 217 [127]. A lag time of oxidation may reduce the apparent reaction rate in the kDPRA for hydroquinones, catechols, and aromatic amines (e.g., p-phenylenediamine).

Acylating agents are poorly recognized by both the KeratinoSens™ and LuSens assays. This is because the sensor protein Keap1 has a cysteine residue for electrophile binding and acylating agents typically transfer their acyl moiety to lysine residues [128]. Other test substances with an exclusive reactivity towards lysine residues will be detected as negative in these assays. Test substances that interfere with the luciferase enzyme can cause either apparent inhibition or increased luminescence (e.g., phytoestrogens) [47]. Substances that interfere with the MTT viability assay (certain dyes and pigments) can be problematic for both assays. An alternative method to assess cell viability, such as the PrestoBlue assay, may be considered [129]. Finally, some chemicals are stressors and can activate the Keap-1/Nrf2/ARE signaling pathway [130] and thus may lead to false positive results (e.g., aspirin, Coumarin) [131,132] even though they are not sensitizers per se.

The endpoint measured in the h-CLAT is the cell-surface expression of CD86 and CD54 determined by flow cytometry using fluorochrome-labeled monoclonal antibodies. As such, strong fluorescent test substances emitting at the same wavelength as fluorescein isothiocyanate (FITC; 530 ± 15 nm) or as propidium iodide (PI; >650 nm) will interfere with the detection and thus cannot be correctly evaluated using FITC-conjugated antibodies or PI. In such cases, alternative fluorochromes or viability markers may be used if equivalency is demonstrated. One additional limitation of the h-CLAT is represented by the lipophilic substances, with an octanol-water partition coefficient (log K_ow_) > 3.5, that may lead to false negatives when compared with LLNA results [110]. Sensitizers present in botanical extracts may be hydrophobic, thus leading to an incorrect classification in the h-CLAT.

Even though some challenges encountered when using cell-based or in chemico assays to address the skin sensitization endpoint have been alleviated by the use of RhE-based NAMs, challenges have been identified for the validated EpiSensA method. For certain chemistries that interfere with the Lactate Dehydrogenase (LDH) assay, such as lactic acid, salicylic acid, glyoxal, imidazolidinyl urea, nanomaterials, etc., an alternative assay is sometimes needed, especially because the LDH assay is used to assess tissue viability in the EpiSensA [133,134,135]. Additionally, elevated expression of the Activating Transcription Factor 3 (ATF3) was reported for several reference non-sensitizers, including glycerol, lactic acid, salicylic acid, and sodium lauryl sulfate [133]. Glycerol also produced false positives for interleukin-8 (IL-8) expression [136]. Still, glycerol and propylene glycol were classified as non-sensitizers, possibly due to non-specific ATF3 induction by low-toxicity, hydrophilic compounds like polyols. Since ATF3 and IL-8 may share regulatory pathways, IL-8 expression could also be triggered by mechanisms unrelated to skin sensitization [137,138]. It is also important to recognize that although RhE-based assays are often considered more robust toward testing undiluted or complex materials, cytotoxicity to the tissue model remains a critical factor. Therefore, the ability to test substances without dilution depends not only on the complexity of the model but also on the compatibility of the test material with the vehicle used and the overall tolerance of the system.

Designing preliminary tests to evaluate potential assay interferences is critical, given the diversity of tested chemistries. Continued industry data sharing on challenging cases is needed to build confidence in NAMs and support the development of fit-for-purpose DAs.

## 5. Strategies and Future Directions

This section highlights several strategies reported on in the last 3–4 years that address challenging materials similar to those analyzed in this manuscript. Several of these emerging technologies combine existing methodologies (some of which are validated) or optimize established procedures to accommodate the difficult-to-test chemical categories.

For instance, it has been shown that a co-culture of keratinocytes (HaCaT) and monocytes (THP-1) triggered secretion of IL-1β and IL-8 following exposure to known haptens such as cobalt (II) chloride (CoCl_2_), methylisothiazolinone (MI), and p-phenylenediamine (PPD) [139]. Another co-culture system combined a reconstructed skin model (SkinEthic^TM^) with the THP-1 cells that are the basis of the validated h-CLAT [140]. Endpoints included CD86 and CD54 (as in h-CLAT), along with CD40 and human leukocyte antigen class II-related subtypes (HLA-DR). The authors concluded that the RhE/THP-1 co-culture may enable assessment of dendritic cell activation by diverse test products, including lipophilic chemicals, mixtures, and finished formulations. Co-culture systems that incorporate both keratinocytes and dendritic cells offer a more physiologically relevant model for assessing skin sensitization compared to traditional monocultures. These models offer greater physiological relevance than monocultures by better replicating immune responses and enabling communication between skin-relevant cells [141].

Another study tested pre-/pro-haptens and lipophilic substances using the RhE model that the EpiSensA method is based upon, combined with in silico tools such as TIMES (TIMES SS; RTSv1) and the OECD QSAR Toolbox (RTSv2) [134]. In a data set of 136 chemicals, RTSv1 supported hazard classification and GHS subcategorization, making it suitable for use in IATA. A noteworthy advantage of RhE skin models is highlighted in a recent study that addressed the challenges in the skin sensitization evaluation of hydrophobic chemicals and mixtures [142]. Their newly developed RhE Nrf2 reporter epidermis model combines the advantages of a reconstructed tissue model and investigates the cytoprotective signaling pathway that is induced by most skin sensitizers and already adopted in OECD 442D assays. A RhE-based Nrf2 reporter model using a Secreted Embryonic Alkaline Phosphatase (SEAP) endpoint showed a 13.5-fold induction with cinnamic aldehyde. Co-exposure with clobetasol propionate suppressed the signal, confirming specificity. Moreover, this test system correctly predicted three sensitizing and two non-sensitizing reference chemicals in a first proof-of-concept study. An advantage of the SEAP endpoint is the cellular secretion of the alkaline phosphatase enzyme into the cell culture medium, which permits the quantification of the reporter gene expression in the supernatant by a simple colorimetric assay rather than by employing sophisticated and long methodologies based on gene expression [143,144,145,146,147]. One current shortcoming of this platform is that the reconstructed tissue model is not commercially available, nor is the test method validated. A key advantage of using RhE models for skin sensitization assessment is their ability to overcome solubility-related limitations, one of the most prominent challenges associated with in chemico and cell-based assays. Compared to cell-based assays, by replicating to a certain extent the structural and barrier properties of human skin, these models enable more realistic exposure conditions and improve compatibility with difficult-to-dissolve substances [148], such as botanical and non-polar device extracts.

Recent attention has focused on human skin explants as the next level of biological complexity [149]. In this study, the skin explant-based test was used to investigate the effects of therapeutic antibodies, which are known to cause adverse reactions in patients. A total of 16 known therapeutic monoclonal antibodies were investigated and the assay was capable of predicting adverse immune reactions (including T cell proliferation and cytokine release) that are implicated in skin sensitization responses to this class of biological products. The availability of this NAM (traded as SkimuneR) is especially important given that, as of 2023, the US FDA no longer requires animal testing for new therapeutics [150]. It is therefore anticipated that ex vivo human disease models will gradually be considered as more relevant platforms to predict adverse effects. This NAM is currently undergoing additional validation through the testing of blinded compounds from industry, where clinical outcomes are known.

Pollutants represent a unique and emerging class of complex mixtures (Table S5). Within this wide chemical space, electronic cigarettes (e-cigarettes) are an increasingly popular alternative to combustible tobacco cigarettes among smokers worldwide [151]. Some natural or synthetic flavors in their composition are known skin allergens. In this study, a GARD™-based testing strategy was used to predict and compare the respiratory and skin sensitizing potential of three experimental and two commercial e-liquids. GARD™skin and GARD™potency differentiated between experimental and commercial e-liquids, classifying the two as weak sensitizers (GHS 1B). Condensates from indoor air were also evaluated for skin sensitization using DPRA and LuSens [152]. Positive DPRA but negative LuSens results suggested potential respiratory sensitization, which is beyond this review’s scope. Formaldehyde is a representative example of a sensitizing compound that shows strong reactivity in the DPRA [42], which is commonly present in indoor as well as outdoor environments [153]. As judged by the limited resources identified, the field of pollutants is emerging and ready for thorough evaluation using modern NAMs that reduce reliance on animal models. 

## 6. Conclusions

**Figure 3:**
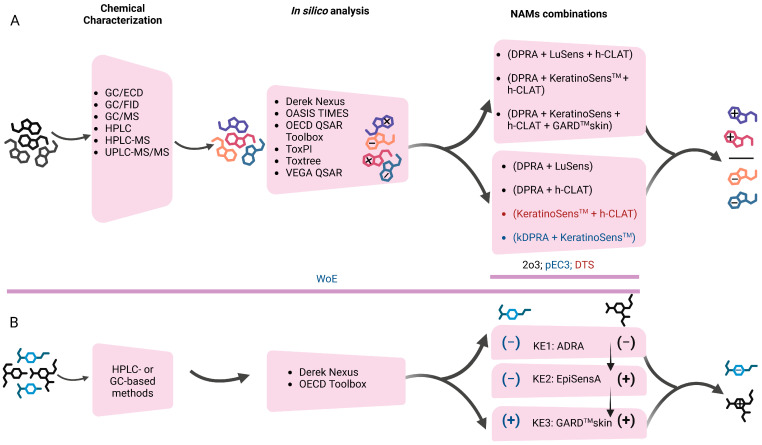
(**A**) Summary of the findings on NAM-based strategies used to assess the skin sensitization potential of complex mixtures, including botanicals, medical and wearable devices, agrochemicals, and pollutants (based on the manuscripts analyzed). The figure outlines typical testing workflows, beginning with chemical characterization and in silico analysis, followed by hazard identification and potency estimation using combinations of validated NAMs. (**B**) Hypothetical approach for consideration of the evaluation of complex mixtures using validated NAMs targeting the first three key events (KEs) in the skin sensitization Adverse Outcome Pathway (AOP). The approach integrates chemical characterization, in silico prediction, and selection of assays (ADRA, EpiSensA, and GARD™skin) that might be used in a combinatorial way to support defined approaches for hazard identification of mixtures. The selection of methods used at each step depends on the product class of interest and existing data to support the methodologies to be used. Abbreviations: 2o3, 2 out of 3 defined approach; ADRA, Amino Acid Derivative Reactivity Assay; DTS, Dermal Sensitization Threshold; DPRA, Direct Peptide Reactivity Assay; EC3 value, the amount of a chemical required to elicit a three-fold increase in LLNA; EpiSensA, Epidermal Sensitization Assay; GARD, Genomic Allergen Rapid Detection; GC/ECD, Gas Chromatography with Electron Capture Detection; GC/FID, Gas Chromatography with Flame Ionization Detection; GC/MS, Gas Chromatography Coupled with Mass Spectrometry; h-CLAT, Human Cell Line Activation Test; HPLC, High-Performance Liquid Chromatography; HPLC-MS, High-Performance Liquid Chromatography Coupled with Mass Spectrometry; kDPRA, kinetic Direct Peptide Reactivity Assay; KE, Key Event; LLNA, Local Lymph Node Assay; OECD, Organization for Economic Cooperation and Development; pEC3, predicted LLNA EC3; QSAR, Quantitative Structure–Activity Relationship; TIMES, Times Metabolism Stimulator for Skin Sensitization; ToxPI, Toxicological Prioritization Index; UPLC-MS/MS, Ultra-Performance Liquid Chromatography coupled with Tandem Mass Spectrometry; VEGA, Virtual models for property Evaluation of chemicals within a Global Architecture; WoE, Weight of Evidence. Created in BioRender. Costin, E. (2025) https://BioRender.com/1ha38pt (accessed 18 August 2025).

Given the complexity of these mixtures and associated challenges, assessing skin sensitization typically requires multiple NAMs targeting different KEs in the AOP. Table 1 and Figure 3A summarize the approaches used and insights gained from applying these strategies to botanicals, medical and wearable devices, agrochemicals, pollutants, and other complex mixtures. In most cases, the test samples begin as chemically undefined or poorly characterized substances. The initial step might involve conducting chemical characterization, even if limited, to guide subsequent analysis. Depending on available composition data and product complexity, in silico tools may provide early hazard insights. Obvious challenges are posed by a lack of information on components’ concentrations or on any synergistic effects that might occur within complex formulations. The next step could involve applying combinations of two to four NAMs to characterize the sensitization hazard. This may be conducted within a DA, such as the 2o3 strategy, to support hazard identification or to determine a Dermal Sensitization Threshold (DTS). Sensitization potency may also be assessed through the derivation of a PoD, such as pEC3 values or ED_01_. Depending on the level of confidence in the individual methodologies, predicting the sensitization potential of complex mixtures may ultimately require a WoE approach that integrates all available data sets, including in silico results.

Based on our analysis of the available literature resources and experience with the application of NAMs for skin sensitization assessment, we can envision a hypothetical approach for evaluating complex mixtures using validated methods (Figure 3B). Although the investigation of analytical techniques is beyond the scope of this review, chromatography-based detection methods, such as HPLC and gas chromatography (GC), have proven reliable for identifying components within complex mixtures. Once the components are identified, the two OECD-adopted in silico tools, Derek Nexus and the OECD QSAR Toolbox, may be used for the early identification of potential sensitizers. To guide the selection of NAMs for generating new data, a reasonable approach would be based on using assays that address the first three KEs in the skin sensitization AOP. At present, no validated NAMs exist for addressing KE4. This strategy provides comprehensive mechanistic insight and supports the application of well-established, defined approaches for hazard identification. For KE1, ADRA provides better solubility compatibility than DPRA or kDPRA [154]. EpiSensA is currently the only validated NAM for KE2 using RhE, whose advantages were discussed in previous sections [105]. GARD™skin is well-suited for KE3, detecting genomic changes even at low concentrations [155]. This is enabled by its flexible solvent options and the assay’s ability to detect genomic activation signatures at low test substance concentrations, improving both sensitivity and applicability for complex mixtures.

This field is continuously expanding and holds significant potential for the development of DAs tailored to the evaluation of complex mixtures and finished products. Advances in this area will help address the limitations currently associated with individual NAMs while taking full advantage of their mechanistic insights. Continued investigation into the application of NAMs across the diverse product categories discussed in this review is essential to ensure broader relevance and reliability. In addition, the development of novel integrated testing strategies that incorporate all available evidence from in silico, in chemico, and in vitro sources will be critical for establishing stronger and more comprehensive DAs. Efforts to validate emerging methods should also remain a priority to enhance regulatory confidence and promote international acceptance. Notably, ongoing technological progress, combined with the collective knowledge generated across industries, is accelerating the shift toward human-relevant skin sensitization assessments without reliance on animal testing.

## Figures and Tables

**Table 1:** Cross-referenced summary on key aspects regarding the mixture categories included in the analysis.

		Botanicals	Extracts of Medical Devices/Wearables	Agrochemicals	Mixtures	Pollutants
	Number of sources analyzed (manuscripts)	11	8	7	9	2
Existing paired data	Human data available	Yes (from clinical studies; no accidental exposure reports)				
	Animal data available	Yes				
NAMs used—summary	Validated methods used	DPRA				
		KeratinoSens™				
		h-CLAT				
			GARD™skin			
		U-SENS™				
		LuSens				LuSens
			kDPRA			
			ADRA			
	RhE-based assay used (validated and non-validated)	Sens-IS			Sens-IS	
			EpiSensA			
	Other non-validated NAMs used	7	h-CLAT (adaptation)	3	4	
	In silico approaches used			OECD QSAR Toolbox		
		Derek Nexus				
					VEGA QSAR	
					Toxtree	
		OASIS TIMES			OASIS TIMES	
	Combinations of validated NAMs used	- h-CLAT + KeratinoSens™ - DPRA + h-CLAT + LuSens - DPRA + h-CLAT + KeratinoSens™	- DPRA + LuSens - kDPRA + KeratinoSens™ - ADRA + EpiSensA + h-CLAT	- GARD™skin + non-validated NAMs - DPRA + h-CLAT + KeratinoSens™	- DPRA + h-CLAT - KeratinoSens™ + h-CLAT - DPRA + h-CLAT + GARD™skin + KeratinoSens™	DPRA + LuSens
NAMs technical aspects—summary	Spiking experiments conducted	Yes				
	Specific challenges identified for the conduct of NAMs	- Botanicals in general—lack of characterization and composition information [54,63]. - Tannins—interference with the assay [15]. - The applicability of in vitro methods is limited for oil-based or cytotoxic botanical extracts [94]. - Certain functional groups of botanical components may fall outside the applicability domain of the methods employed [17].	- Non-polar extracts are poorly soluble and may not be able to be assessed in the in vitro assays. - A weak sensitizer can be mispredicted when the extraction ratio is not sufficiently high [106]. - Devices containing metals may be under-predicted in the DPRA [107].	- Assumed molecular weight used for calculation of test substance concentrations [111].	- DPRA prediction is possible if sensitizers are more than 1 potency level apart [39]. - DPRA may not be suitable for chemicals that are pro-haptens [68]. - The DPRA test method can be suitable for use with well-characterized mixtures [74].	Method(s) used for sampling the pollutants can vary and affect the results. Assessing indoor air pollution based solely on individual chemicals is not an adequate approach [152].
	Unique aspects discussed	- Establishment of botanicals reference set [95]. - B-PPRA incorporates an oxidation system (+HRP/P) for enhanced identification of potential pre- and pro-haptens [86].	- DNCB spiked in polyurethane sheets identified as a useful positive control [108]. - Use of human artificial sweat as extraction solvent [7]. - NAMs can be used for risk assessment of devices by testing the device neat components [104].	- GARD™skin assay coupled to proteomic analysis of treated cells [48]. - Potency classification using the GARD™potency assay [114].	- DST established for mixtures negative in KeratinoSens™ + h-CLAT [70]. - Evaluation of the vehicle type on the prediction of skin sensitization for RhE models [71].	Potency classification using the GARD™potency assay [151].
Defined approaches	Potency addressed	Yes [95]	Yes [104]	Yes [5,46,114]	Yes [68,71]	Yes [152]
	Strategies (DA, WoE, others)	- WoE [95] - 2o3 [17]	- pEC3 [104] - 2o3 [103]	- 2o3 [5] - WoE + animal data [113]	- DST [70] - WoE (combination of 4 validated assays + in silico model) [71]	2o3 [152]

Abbreviations: 2o3, 2 out of 3 approach; ADRA, Amino Acid Derivative Reactivity Assay; BrdU, Bromodeoxyuridine; DA, Defined Approach; DNCB, 2,4-dinitrochlorobenzene; DPRA, Direct Peptide Reactivity Assay; DST, Dermal Sensitization Threshold; EC3 value, the amount of a chemical required to elicit a three-fold increase in LLNA (Local Lymph Node Assay); GARD, Genomic Allergen Rapid Detection; h-CLAT, Human Cell Line Activation Test; kDPRA, kinetic Direct Peptide Reactivity Assay; NAM, New Approach Methodology; OECD, Organization for Economic Co-operation and Development; pEC3, predicted EC3 value; RhE, reconstructed human epidermis; QSAR, Quantitative Structure–Activity Relationship; TIMES, Times Metabolism Stimulator for Skin Sensitization; WoE, Weight of Evidence. Note: The references are presented in chronological order and alphabetically within the same year (where applicable).

## Data Availability

No new data were created or analyzed in this study. Data sharing is not applicable to this article.

## Supplementary Materials

The following supporting information can be downloaded at: https://www.mdpi.com/article/10.3390/toxics13080693/s1, Table S1. New Approach Methodologies used to assess the skin sensitization potential of mixtures. Table S2. New Approach Methodologies used to assess the skin sensitization potential of botanicals. Table S3. New Approach Methodologies used to assess the skin sensitization potential of medical and wearables devices. Table S4. New Approach Methodologies used to assess the skin sensitization potential of agrochemicals. Table S5. New Approach Methodologies used to assess the skin sensitization potential of pollutants.
