# Supplementary material for: New Horizons in Skin Sensitization Assessment of Complex Mixtures: The Use of New Approach Methodologies Beyond Regulatory Approaches
Source: Toxics. 2025 Aug 20;13(8):693. doi: 10.3390/toxics13080693 (PMC12390330; doi:10.3390/toxics13080693)
Supplement: Supplementary file 1 [file toxics-13-00693-s001.zip › Table S1.pdf]

Table S1. New Approach Methodologies used to assess the skin sensitization potential of mixtures.

Testing Methodology		Mixtures Specifics		Conclusions	Reference
NAM Test System	Paired *In Vivo*/Other Data	Type of Mixtures Tested	Intended Use and Product Type		
DPRA	LLNA	Mixture of aldehydes:<br>- Hydroxycitronellal-Citral (two weak sensitizers together)<br>- Citral-Cinnamaldehyde (pair of weak-moderate sensitizers)	Perfumes	- All 3 aldehydes belong to the 26 fragrance ingredients list (Annex 3 Cosmetics Directive 76/768/EEC) identifying sensitizers that need to be labelled on the packaging of cosmetic products.<br>- DPRA detected the presence of sensitizers in the mixtures. In general, the most reactive chemical in each mixture was described as the strongest sensitizer in LLNA and DPRA. It could be hypothesized that the DPRA can estimate the chemical reactivity of the mixture similar to that of the stronger sensitizer component.<br>- Citral was much more reactive in the mixture than hydroxycitronellal, but it reacted more with Lys than with Cys. The DPRA depletion of both peptides would then be overrepresented and underestimated, respectively.<br>- When in mixture, even if cinnamaldehyde was more reactive with Cys than citral, both compounds reacted enough with Lys. Thus, the reactivity of the mixture resulted from the sum of both reactivities.<br>- The assessment of mixtures' reactivity by using the DPRA could be attempted if one component is much more reactive and thus more strongly sensitizing than the other (*i.e.*, strong against weak sensitizer). However, a prediction becomes difficult when components are of close sensitizing potential such as fragrance aldehydes (weak or moderate), which react towards Lys.	[39]
- DPRA<br>- h-CLAT	- LLNA<br>- Human data	Mixture of:<br>- Citral (fragrance)<br>- DDAC (antimicrobial)	- Biocides<br>- Household deodorants	- In three independent h-CLAT experiments, DDAC and citral were predicted to be sensitizers, while ethylene glycol was predicted to be a non-sensitizer.<br>- Mixtures of DDAC or citral with ethylene glycol at 7:3 and 1:4 (w/v) ratios, respectively, tested positive for skin sensitization potential in the h-CLAT	[68]

Testing Methodology		Mixtures Specifics		Conclusions	Reference
NAM Test System	Paired *In Vivo*/Other Data	Type of Mixtures Tested	Intended Use and Product Type		
		- Ethylene glycol (excipient)		assay. However, the sensitization potency decreased as the proportion of ethylene glycol increased.<br>- Citral and its mixtures with ethylene glycol were all positive, while DDAC and its ethylene glycol mixtures were all negative by the DPRA. The results indicate that the DPRA method may not suitable for chemicals with pro-hapten characteristics.	
- h-CLAT<br>- KeratinoSens™	LLNA	No products or chemicals were tested. Database of 146 chemicals with existing h-CLAT, Keratinosens™, and LLNA data was used for interpretations.	NA	- Some skin sensitizers showed higher detection limits in the binary battery of *in vitro* test methods (KeratinoSens™ and h-CLAT) than LLNA. To minimize the uncertainty associated with decreased sensitivity for this type of sensitizers, a risk assessment strategy was developed for mixtures with negative results from this test battery. The assumption was that a NESIL can be derived for mixtures with negative *in vitro* test results.<br>- A total of 146 sensitizers with *in vitro* and LLNA data according to the assumption of indeterminate constituents in final mixtures were analyzed.<br>- A probabilistic assessment using 95th percentiles of calculated NESILs for mixtures (mixture NESILs) was performed and a DST of 6010 μg/cm^2 was derived for mixtures that were negative in the binary test battery.<br>- This threshold represents an exposure level below which mixtures that tested negative in the KeratinoSens™ and h-CLAT assays are unlikely to induce skin sensitization. This approach can be applied to other mixtures of unknown composition by using the derived DST as a screening-level safety margin. It offers a non-animal, alternative to traditional risk assessment, especially valuable when detailed compositional analysis is unfeasible.	[70]

Testing Methodology		Mixtures Specifics		Conclusions	Reference
NAM Test System	Paired *In Vivo*/Other Data	Type of Mixtures Tested	Intended Use and Product Type		
Sens-IS	LLNA historical data on ingredients	- 3 chemical allergens (cinammaldehyde, hydroxycitronellal, isoeugenol) prepared in 5 different cosmetic formulation types (oil, water in oil, oil in water, cleansing water and microemulsion)<br>- 2 fragrance blends<br>- 25 commercial cosmetic and skin care products	- Cosmetic<br>- Skin care products	- The impact of the vehicle on the absorption of individual ingredients through the reconstructed tissue model was evaluated by testing the sensitization potential of commonly used cosmetic formulation types containing three chemical allergens and two fragrance blends.<br>- The sensitization potential of the three allergens was significantly reduced when tested in microemulsion while the cleansing water preparation significantly increased it. Water in oil, oil in water or oil preparations had significant but more moderate (enhancing or reducing) effects on the skin sensitization potency of the tested chemicals.<br>- The influence of irritants (SDS and Lactic acid) on the sensitizing potency of various allergens was also tested. The Sens-IS assay detected an enhancement of the potency of some allergens when mixed with non-irritating concentrations of irritant chemicals.<br>- The study also investigated the impact of combining different sensitizers to assess how mixtures influence the sensitization threshold. Notably, some chemical combinations that were non-reactive when tested individually, produced a positive response in the Sens-IS assay when mixed, indicating a potential synergistic effect.<br>- A total of 21 out of the 25 finished cosmetic product tested were determined to be non-irritant and non-sensitizers.	[71]
KeratinoSens™	Historical Data:<br>- GMPT	Mixtures of:<br>- Allergens:<br>• Cinnamal	- NA	- When skin allergens were combined with irritants, the sensitization thresholds generally decreased, meaning that lower concentrations of allergens were sufficient to elicit a sensitization response.	[75]

Testing Methodology		Mixtures Specifics		Conclusions	Reference
NAM Test System	Paired *In Vivo*/Other Data	Type of Mixtures Tested	Intended Use and Product Type		
	- LLNA	• EGDMA<br>- Irritants:<br>• SDS<br>• Salicylic acid<br>• α-pinene		- The nature of the allergen played a dominant role in the outcome of the mixture. In particular, the weaker sensitizer (EGDMA) showed more pronounced synergistic effects when combined with irritants, especially with SDS.<br>- The KeratinoSens™ assay proved to be a valuable tool for quantitatively detecting and comparing sensitization responses in mixtures, making it a promising option for improving skin sensitization assessment without animal testing.	
h-CLAT	LLNA:BrdU-FCM	Mixtures of:<br>- PG<br>- PHMG<br>- TCS	Household products (humidifier disinfectant)	- PG is a non-sensitizer by LLNA, KeratinoSens™ and U-SENS™, but is considered allergenic by epidemiological and human case studies.<br>- PHMG and TCS are considered moderate sensitizers based on LLNA.<br>- The skin sensitization potentials of PHMG or TCS or their mixtures with PG have not been previously investigated by the use of alternative test methods.<br>- Using the h-CLAT assay, all the three substances were predicted to be sensitizers, and mixtures of PHMG or TCS with PG at ratios of 9:1, 4:1, or 1:4 (w/v) were all positive in terms of skin sensitization potential.	[73]
DPRA	LLNA	9 individual chemicals were first tested (6 sensitizers and 3 non-sensitizers)<br><br>Mixtures of the chemicals below were tested in combination:	- Consumer products<br>- Cosmetics	- The DPRA's predictive capability for individual substances was initially assessed. The selected chemicals were tested based on their LLNA EC3 values, which covered a concentration range from 0.46 mM to nearly 2000 mM, which deviates from the recommended 100 mM stipulated in the test guideline. However, mixtures were prepared with each component at the 100 mM concentration.	[74]

Testing Methodology		Mixtures Specifics		Conclusions	Reference
**NAM Test System**	**Paired *In Vivo*/Other Data**	**Type of Mixtures Tested**	**Intended Use and Product Type**		
		- 1-Butanol (non-sensitizer)<br>- 2,3-Butanedione (weak sensitizer)<br>- 2,4-Dinitrochlorobenzene (extreme sensitizer)<br>- Benzylideneacetone (moderate sensitizer)<br>- Farnesal (weak sensitizer)<br>- Formaldehyde (strong sensitizer)<br>- Lactic acid (non-sensitizer)<br>- Oxazolone (extreme sensitizer)		- One extremely potent sensitizer, oxazolone, was incorrectly classified as a non-sensitizer when tested at its EC3 of 0.4 mM instead of the DPRA standard concentration.<br>- The applicability of the DPRA to test unknown mixtures was assessed. In these experiments, the complexity of unknown mixtures was reduced to mixtures containing either two known skin sensitizers with varying potencies, or a combination of a skin sensitizer with a non-skin sensitizer, or multiple non-sensitizers.<br>- For binary mixtures, the DPRA was able to distinguish all sensitizers, while the strongest sensitizer in the mixture was determinant for the overall observed peptide depletion.<br>- Overall, the DPRA test method can be used efficiently for well-known characterized mixtures. However, when deviating from the recommended testing concentration of 100 mM, caution should be taken in case of negative results, limiting the DPRA's applicability for mixtures.	
- HaCaT assay<br>- OECD QSAR Toolbox<br>- RhE (Epi-Derm™) assay<br>- THP-1 assay<br>- Toxtree<br>- VEGA QSAR	LLNA for discrete compound of DiPeP	- DiPeP may exist either as a mixture consisting of several branched positional isomers or as single defined structure, depending upon the nature of the alcohol used in its synthesis.<br>- DiPeP is considered an UVCB substance.	Plasticizer or additive within the production of PVC	- *In silico* predictions did not identify DiPeP as a skin sensitizer when evaluated as a discrete compound.<br>- The results obtained using the HaCaT and RhE assays were discordant. While the HaCaT assay showed that DiPeP can activate keratinocytes (increased levels of IL-6, IL-8, IL-1α, and *ILA* gene expression), in the RhE assay DiPeP slightly increased IL-6 release.<br>- The role of DiPeP in KE3 (dendritic cell activation) was demonstrated by the increased levels of CD54, IL-8 and TNF-α in THP-1 cells (THP-1 activation assay).	[72]

Testing Methodology		Mixtures Specifics		Conclusions	Reference
NAM Test System	Paired *In Vivo*/Other Data	Type of Mixtures Tested	Intended Use and Product Type		
				- Overall, the findings were inconclusive regarding the skin sensitization potential of DiPeP, contrasting with the results obtained from the LLNA.	
- DPRA<br>- GARD™skin<br>- h-CLAT<br>- KeratinoSens™<br>- OASIS TIMES<br>- OECD QSAR Toolbox	LLNA	- 2 poorly soluble substances [Tetrakis (2-ethylbutyl) orthosilicate] and Decyl palmitate<br>- 2 UVCB substances [Alkylated anisole and hydrazinecarboximidamide, 2-[(2-hydroxyphenyl)methylene]-, reaction products with 2 undecanone]	Petrochemical industry products	- Tetrakis (2-ethylbutyl) orthosilicate was determined to be unlikely to have skin sensitization potential by WoE (inconclusive based on QSAR and DPRA due to technical limitations; negative in h-CLAT with low confidence but negative with high confidence in GARD™skin; positive in LLNA).<br>- Hydrazinecarboximidamide, 2-[(2-hydroxyphenyl)methylene]-, reaction products with 2 undecanone was determined to be inconclusive by WoE (structural alerts for 1/3 of the hypothesized parent structures; TIMES with autoxidation model predicted a positive result for 1/3 of the hypothetical structures, but inadequate since the structures fell outside of the model applicability domain; DPRA not applicable; negative in KeratinoSens™; positive in h-CLAT; GARD™skin predicted it a sensitizer; moderate sensitizer in LLNA).<br>- Decyl palmitate is unlikely to be a skin sensitizer, as supported by *in silico* analyses and a GARD™skin prediction, both classifying it as a non-sensitizer. LLNA results were borderline, and thus associated with low confidence of the prediction.<br>- Alkylated anisole is unlikely to be a skin sensitizer based on the WoE. Despite conflicting results from negative *in silico* predictions and positive *in vivo* data, GARD™skin classified it as a non-sensitizer.	[69]

BrdU, Bromodeoxyuridine; CD54, Cluster of Differentiation 54, protein encoded by the Intercellular Adhesion Molecule 1; Cys, Cysteine; DDAC, didecyldimethylammonium chloride; DiPeP, diisopentyl phthalate; DPRA, Direct Peptide Reactivity Assay; DST, Dermal Sensitization Threshold; EC3 value, the amount of a chemical required to elicit a three-fold increase in LLNA; EGDMA, ethylene glycol dimethacrylate; FCM, Flow Cytometry Method; GARD, Genomic Allergen Rapid Detection; h-CLAT, human Cell Line Activation Test; IL, Interleukin; KE, Key Event; LLNA, Local Lymph Node Assay; Lys, Lysine; NA,

Not Applicable; NAM, New Approach Methodology; NESIL, No Expected Sensitization Induction Level; OECD, Organization for Economic Co-operation and Development; PG, propylene glycol; PHMG, polyhexamethylene guanidine; PVC, polyvinyl chloride; RhE, Reconstructed human Epidermis; QSAR, Quantitative Structure-Activity Relationship; SDS, Sodium Dodecyl Sulfate; TCS, triclosan; TIMES, Times Metabolism Stimulator for Skin Sensitization; TNF, Tumor Necrosis Factor; UVCB, unknown or variable composition complex mixture reaction products or biological materials; VEGA, Virtual models for property Evaluation of chemicals within a Global Architecture; WoE, Weight of Evidence.

Note: The references are presented in chronological order and alphabetically within the same year (where applicable).
