# Supplementary material for: New Horizons in Skin Sensitization Assessment of Complex Mixtures: The Use of New Approach Methodologies Beyond Regulatory Approaches
Source: Toxics. 2025 Aug 20;13(8):693. doi: 10.3390/toxics13080693 (PMC12390330; doi:10.3390/toxics13080693)
Supplement: Supplementary file 1 [file toxics-13-00693-s001.zip › Table S2.pdf]

Table S2. New Approach Methodologies used to assess the skin sensitization potential of botanicals.

Testing Methodology		Botanical Specifics					Conclusions	Reference
NAM Test System	Paired *In Vivo*/Other Data	Plant Tested (Part of Plant Extracted)	Type of Extract Preparation	Chemical Characterization Method	Extracted Ingredient Identified with Sensitization Potential	Intended Use/Product Type		
KeratinoSens™	NA	- Chamomile (flower)<br>- Green Tea (leaf)<br>- Gurana (seed)<br>- Papaya (fruit)	- Chamomile: flower solubilized in 27% propylene glycol and 53% water<br>- Green Tea: powder<br>- Gurana and Papaya: fruit and seed, respectively, solubilized in 2.5% water and 96.8% glycerol as carrier<br><br>Extracts were spiked with different doses of known sensitizers:<br>- Cinannmic aldehyde<br>- Citral<br>- Isoeugenol	NP	- Chamomile: 20% active plant matter consisted mostly in flavonoids<br>- Green Tea: 95% polyphenols and 50% catechin<br>- Gurana and Papaya: 0.7% of active plant content	Cosmetic product	- Based on LLNA data for the sensitizers used in the spiked extracts, citral is not expected to be detected when present at concentrations below 10% in mixtures, while cinnamic aldehyde and isoeugenol are not expected to be detected below 2% and 1.5%, respectively.<br>- The results indicate that a single moderate sensitizer can be detected when added to non-cytotoxic plant extracts that are otherwise negative in the KeratinoSens™ assay. Detection of isoeugenol at 5% and citral at 0.5% in the spiked chamomile extract highlights the sensitivity of this NAM.	[14]
- HaCaT IL-18<br>- mDPRA<br>- MUSST IL-8	NA	*Eugenia dysenterica* (leaf)	Hydroalcoholic EDE (2.5-1,000 µg/mL)	HPLC	- Tannin (ellagic acid)	Cosmetic product	- mDPRA: EDE was correctly classified as a skin sensitizer in the high reactivity class. Cysteine depletion was particularly pronounced and showed	[15]

Testing Methodology		Botanical Specifics					Conclusions	Reference
NAM Test System	Paired *In Vivo*/Other Data	Plant Tested (Part of Plant Extracted)	Type of Extract Preparation	Chemical Characterization Method	Extracted Ingredient Identified with Sensitization Potential	Intended Use/Product Type		
					- Flavonoids (quercetin and gallic acid)		a concentration-dependent pattern. Based on mean peptide depletion, EDE fell within the moderate to high reactivity category.<br>- HaCaT and IL-18 assay: EDE did not induce an increase in IL-18 levels in HaCaT cells at non-cytotoxic concentrations of 40, 80, and 160 µg/mL, indicating no sensitization potential for this endpoint.<br>- MUSST and IL-8 assay: EDE was classified as a non-sensitizer at non-cytotoxic concentrations ranging from 12.5 to 200 µg/mL. Tannins are known to form complexes with proteins through hydrogen bonding and hydrophobic interactions, which may partially interfere with the analytical detection of peptide depletion induced by EDE.	

Testing Methodology		Botanical Specifics					Conclusions	Reference
NAM Test System	Paired *In Vivo*/Other Data	Plant Tested (Part of Plant Extracted)	Type of Extract Preparation	Chemical Characterization Method	Extracted Ingredient Identified with Sensitization Potential	Intended Use/Product Type		
DCYA	NA	- *Calendula officinalis* (flower)<br>- *Cinnamonum verum* (bark)<br>- *Magnolia grandiflora* (leaf)<br>- *Rosa canina* (fruit)	- *Cinnamonum verum* and *Calendula officinalis* extracted in methanol<br>- *Magnolia grandiflora* was used to select the solvent from methanol, acetone, diethyl ether, ethyl acetate, cyclohexane and hexane<br>- *Rosa canina* extracted in methanol<br><br>The least DCYA-reactive extract (of *Rosa canina*) was spiked with the following sensitizers at various concentrations:<br>- Cinnamaldehyde<br>- Coumarin<br>- Parthenolide	NP	- *Calendula officinalis*: no sensitizing components<br>- *Cinnamonum verum*: cynnamaldehide<br>- *Magnolia grandiflora*: sesquiterpene lactones (parthenolide, costunolide)<br>- *Rosa canina*: no sensitizing components	NS	- The extract of *Rosa canina* was spiked with parthenolide, cinnamaldehyde, and coumarin, each prepared as a 20 mM stock. The final spiking concentrations were selected to range from 50% to 150% of the optimal concentration (5 mM) for pure compounds (3 concentrations were used).<br>- The methanol extract of *Rosa canina* spiked with parthenolide showed measurable DCYA depletion (TRI increase) at all tested concentrations. When the plant material was spiked with 1 equivalent of parthenolide, a concentration-dependent reactivity was evident between 5 and 20 mg/mL of total extract. A saturated response occurred when the parthenolide/DCYA ratio exceeded	[18]

Testing Methodology		Botanical Specifics					Conclusions	Reference
NAM Test System	Paired *In Vivo*/Other Data	Plant Tested (Part of Plant Extracted)	Type of Extract Preparation	Chemical Characterization Method	Extracted Ingredient Identified with Sensitization Potential	Intended Use/Product Type		
							2:1, resulting in complete DCYA depletion at all tested extract concentrations.<br>- *Rosa canina* samples spiked with the moderate sensitizer, cinnamaldehyde, produced a concentration-dependent fluorescence response, although only partial DCYA reactivity was observed at any tested concentration.<br>- In contrast, no concentration-dependent reactivity was observed in the presence of coumarin, a non-reactive electrophile.	
- DPRA<br>- h-CLAT<br>- KeratinoSens™<br>- Sens-IS<br>- U-SENS™	- LLNA<br>- Human	- *Jasmine absolute frandiflorum*<br>- *Jasmine absolute sambac*<br>- Oakmoss<br>- Treemoss	NP	NP	NA	NA	- Natural extracts present specific challenges, as their composition depends on the source material and extraction process. As a result, they cannot be precisely defined and are likely to vary over time. This variability compromises comparisons with	[54,63]

Testing Methodology		Botanical Specifics					Conclusions	Reference
NAM Test System	Paired *In Vivo*/Other Data	Plant Tested (Part of Plant Extracted)	Type of Extract Preparation	Chemical Characterization Method	Extracted Ingredient Identified with Sensitization Potential	Intended Use/Product Type		
		- Tea leaf absolute<br>- Ylang-ylang					existing data, which often lack adequate documentation of extract composition.<br>- A comprehensive, harmonized database of 128 substances was generated and included results from five NAMs, LLNA and human reference data. This group included 6 natural extracts, providing diversity on the data set to be used as reference covering various domains such as complex mixtures.	
- h-CLAT<br>- KeratinoSens™	LLNA	NA	NA	NA	NA	Cosmetics	- This study was based on the assumption that botanical extracts may contain skin sensitizers that yield positive results in *in vitro* test methods. The detection limits of these sensitizers in botanical extracts were compared between the LLNA and the in vitro assays KeratinoSens™ and h-CLAT.	[94]

Testing Methodology		Botanical Specifics					Conclusions	Reference
**NAM Test System**	**Paired *In Vivo*/Other Data**	**Plant Tested (Part of Plant Extracted)**	**Type of Extract Preparation**	**Chemical Characterization Method**	**Extracted Ingredient Identified with Sensitization Potential**	**Intended Use/Product Type**		
							- It was assumed that the botanical extracts were water-soluble, non-cytotoxic, and did not exhibit general masking effects that could interfere with the detection of skin sensitizers in their composition.<br>- A total of 146 chemicals were analyzed, of which 116 and 129 were positive in the KeratinoSens™ and h-CLAT assays, respectively.<br>- The results support the broad applicability of *in vitro* assays for detecting skin-sensitizing constituents in botanical extracts. However, the applicability of *in vitro* methods is limited for oil-based or cytotoxic botanical extracts.<br>- Some sensitizers showed higher detection limits on *in vitro* test methods than LLNA, suggesting that the botanical extract containing sensitizers	

Testing Methodology		Botanical Specifics					Conclusions	Reference
NAM Test System	Paired *In Vivo*/Other Data	Plant Tested (Part of Plant Extracted)	Type of Extract Preparation	Chemical Characterization Method	Extracted Ingredient Identified with Sensitization Potential	Intended Use/Product Type		
							might be positive in LLNA but negative in the binary test battery.	
- h-CLAT<br>- h-CLAT adaptation in DC2.4 cells	LLNA	Brazilian Green Propolis (from *Baccharis dracunculifolia*)	95% Ethanol/water extraction (55% BGP in ethanol extract was tested)	Provided by the extract supplier	Cinnamic acid derivatives	- Biopharmaceuticals<br>- Cosmetics<br>- External preparations<br>- Food supplements<br>- Shampoos<br>- Toothpaste<br>- Traditional medicine	- BGP was identified as a non-sensitizer in the standard h-CLAT assay.<br>- BGP was determined as positive in the DC2.4 adaptation of the h-CLAT assay, and was identified as a moderate skin sensitizer in the LLNA.<br>- The appropriate classification of BPG when using the DC2.4 adaptation of the h-CLAT assay indicates that the proposed model may have increased sensitivity for the detection of sensitizers that otherwise may lead to false negative results in the standard h-CLAT assay.	[4]
DPRA	NA	- *Lavandula angustifolia*<br>- *Melissa officinalis*<br>- *Mentha longifolia*	NP	GC/MS	NP	Cosmetics	- The DPRA was able to predict 4 out of 6 essential oils as skin sensitizers. These results appear to be in accordance with literature reports regarding the potential of these oils to cause skin sensitization.	[93]

Testing Methodology		Botanical Specifics					Conclusions	Reference
NAM Test System	Paired *In Vivo*/Other Data	Plant Tested (Part of Plant Extracted)	Type of Extract Preparation	Chemical Characterization Method	Extracted Ingredient Identified with Sensitization Potential	Intended Use/Product Type		
		- *Rosmarinus officinalis*<br>- *Salvia officinalis*<br>- *Thymus vulgaris*					- Based on these results, the authors propose DPRA as an assay capable to identify the sensitization potential of essential oils.	
- B-PPRA<br>- Derek Nexus<br>- OASIS TIMES *in silico* model	NA	- *Arnica montana* (flower)<br>- Calendula (flower)<br>- Chamomile (flower)<br>- Feverfew (leaf)<br>- Propolis (bee glue)<br>- Rosemary (leaf)	The botanical extracts contained <2% active in a carrier composed of 15-25% water and either 75-85% glycerin or 75-85% propylene glycol.<br><br>Sensitivity of B-PPRA was evaluated using the Chamomile extract (85% propylene glycol and 15% water) spiked with:	Multiple as identified through literature search<br><br>(*see the Discussion section in the manuscript for more details*)	The most likely components with sensitization potential were identified based on literature search and by *in silico* determination<br><br>(*see the Discussion section in the manuscript for more details*)	Consumer products	- A modification of PPRA was explored in this study for screening of botanicals or natural substances. The assay incorporates an oxidation system (+HRP/P) for the activation of potential pre- and pro-haptens.<br>- Initial studies demonstrated that B-PPRA results were reproducible and the carrier's impact was low.<br>- The spiking experiments identified mixtures containing isoeugenol as sensitizers at concentrations as low as 0.05%.<br>- The presence of pro- or pro-hapten constituents contribute to the higher	[86]

Testing Methodology		Botanical Specifics					Conclusions	Reference
NAM Test System	Paired *In Vivo*/Other Data	Plant Tested (Part of Plant Extracted)	Type of Extract Preparation	Chemical Characterization Method	Extracted Ingredient Identified with Sensitization Potential	Intended Use/Product Type		
			- Cinnamic aldehyde (moderate sensitizer that forms adducts and oxidizes Cys to form dimers)<br>- Citral (weak sensitizer that forms adducts in peptide reactivity assays)<br>- Isoeugenol (moderate sensitizer that forms adducts and oxidizes Cys to form dimers, is classified as a pre-hapten)				peptide depletion observed for extracts of *Arnica montana*, Calendula, Propolis, and Rosemary and vice-versa for extracts of Feverfew and Chamomile which predicted to be non-sensitizers.<br>- B-PPRA may be considered as a screening assay and used as part of an integrated approach for skin sensitization risk assessment.	
- DPRA<br>- h-CLAT<br>- LuSens<br>- mMUSST<br>- Sens-IS	- LLNA<br>- GPMT	- *Bixa orellana*<br>- *Cariniana brasiliensis*<br>- *Cistus monspeliensis*<br>- *Diospyros mespliformis*	Commercial plant extracts tested as follows:<br>- DPRA: 8 extracts<br>- h-CLAT: 8 extracts<br>- LuSens: 4 extracts<br>- mMUSST: 2 extracts	NA	NA	- Nutrition products<br>- Personal care products	- The balanced accuracy was 50% for DPRA, h-CLAT, and mMUSST; 67% for LuSens, and 88% for Sens-IS.<br>- The 2o3 approach was also evaluated for some of the extracts with available DPRA, LuSens, and h-CLAT data, resulting in a balanced accuracy of 50%.	[17]

Testing Methodology		Botanical Specifics					Conclusions	Reference
NAM Test System	Paired *In Vivo*/Other Data	Plant Tested (Part of Plant Extracted)	Type of Extract Preparation	Chemical Characterization Method	Extracted Ingredient Identified with Sensitization Potential	Intended Use/Product Type		
		- *Epilobium angustifolium*<br>- *Ferula hermonis*<br>- *Grifola frondosa*<br>- *Hippophae rhamnoides*<br>- *Lansium domesticum*<br>- *Manilkara multinervis*<br>- *Parkia biglosa*<br>- *Persicaria bistorta*	- Sens-IS: 12 extracts<br><br>(*see the manuscript for more details on the identity of extracts tested and using which assays)*				- When mMUSST was used within the 2o3 approach instead of h-CLAT, turned one true-positive extract (*Parkia biglosa*) into a false-negative, while *Cariniana brasiliensis* remained a true-positive.<br>- The authors indicate that the 2o3 approach that includes DPRA, LuSens/KeratinoSens™, and h-CLAT is not recommended for botanical extracts.<br>- The results showed that some substance subgroups may not be within the applicability domains of the methods used.	
- DPRA<br>- h-CLAT<br>- Keratinosens™<br>- Sens-IS	- GPMT<br>- HMT<br>- HRIPT<br>- LLNA	- *Aloe barbadensis* (inner leaf gel)<br>- *Allium sativum* (bulb)	Varied depending on the plant, part of the plant, etc.<br><br>(*see details included in the manuscript for each of the botanicals used*)	Accomplished through literature search for each botanical extract	For each botanical, literature information on composition and drivers for	- Consumer products<br>- Cosmetics<br>- Household care products	- This study compiled the available information for 14 commonly used botanicals in consumer products, to classify their sensitization potential using a WoE approach.<br>- Based on available information, the botanicals were classified into:	[95]

Testing Methodology		Botanical Specifics					Conclusions	Reference
NAM Test System	Paired *In Vivo*/Other Data	Plant Tested (Part of Plant Extracted)	Type of Extract Preparation	Chemical Characterization Method	Extracted Ingredient Identified with Sensitization Potential	Intended Use/Product Type		
		- *Anacardium occidentale* (nutshell)<br>- *Camellia sinensis* (leaf)<br>- *Centella asiatica* (whole plant)<br>- *Citrullus lanatus* (seed oil)<br>- *Cucumis sativus* (fruit)<br>- *Matricaria chamomila* (flower)<br>- *Rhodymenia palmata* (whole algae)<br>- *Rosmarinus officinalis* (leaf)<br>- *Silybum marianum* (fruit, seed)		(*see the manuscript for specific details*)	sensitization effect is included<br><br>(*see the manuscript for specific details on each extract*)		a. Strong evidence to cause sensitization<br>b. Some but limited evidence of causing sensitization<br>c. Strong evidence that they are non-sensitizers<br>- When available data include sufficient potency-related information, a sensitization potency assessment was also provided using a WoE approach, classifying the botanicals as strong, moderate, weak, or non-sensitizers..<br>- The proposed framework include the following sequential steps:<br>1. Determine if the exposure to the botanical is below the threshold of exposure; if not, proceed to step 2<br>2. Compile all available historical information; if the information is insufficient to classify the hazard of the	

Testing Methodology		Botanical Specifics					Conclusions	Reference
NAM Test System	Paired *In Vivo*/Other Data	Plant Tested (Part of Plant Extracted)	Type of Extract Preparation	Chemical Characterization Method	Extracted Ingredient Identified with Sensitization Potential	Intended Use/Product Type		
		- *Tanacetum parthenium* (leaf, petal)<br>- *Toxicodendron diversilobum* (leaf, stem, bark, fruit)<br>- *Toxicodendron radicans* (leaf, stem, fruit)					botanical, proceed to step 3;<br>3. Generate new data, such as NAMs data, then return to 2.<br>- The set of botanicals included in this analysis can be considered a reference set based on their "data rich" profile covering a range of sensitization potencies that can be used to evaluate existing test methods or support the development of new predictive models for skin sensitization.	

2o3, 2 out of 3 approach; BGP, Brazilian Green Propolis; B-PPRA, Botanicals Peroxidase Peptide Reactivity Assay; Cys, Cysteine; DC, dendritic cells; DCYA, Dansyl Cysteamine Assay; DPRA, Direct Peptide Reactivity Assay; EDE, *Eugenia dysenterica* extract; DCYA, Dansyl Cystemine Assay; GC/MS, Gas Chromatography/Mass Spectrometry; GPMT, Guinea Pig Maximization Test; h-CLAT, human Cell Line Activation Test; HMT, Human Maximisation Test; HPLC, High-Performance Liquid Chromatography; HRIPT, Human Repeated Insult Patch Test; HRP/P, Horseradish Peroxidase/Hydrogen peroxide; IL, Interleukin; LLNA, Local Lymph Node Assay; mDPRA, micro or modified DPRA; mMUSST, modified Myeloid U937 Skin Sensitization Test; MW, Molecular Weight; NA, Not Applicable; NAM, New Approach Methodology; NP, Not Provided; NS, Not Specified; TIMES, Times Metabolism Stimulator for Skin Sensitization; TRI, Total Reactive Index; WoE, Weight of Evidence.

Note: The references are presented in chronological order and alphabetically within the same year (where applicable).
