# Supplementary material for: New Horizons in Skin Sensitization Assessment of Complex Mixtures: The Use of New Approach Methodologies Beyond Regulatory Approaches
Source: Toxics. 2025 Aug 20;13(8):693. doi: 10.3390/toxics13080693 (PMC12390330; doi:10.3390/toxics13080693)
Supplement: Supplementary file 1 [file toxics-13-00693-s001.zip › Table S3.pdf]

Table S3. New Approach Methodologies used to assess the skin sensitization potential of medical and wearables devices.

Testing Methodologies		Medical Devices and Wearables Specifics				Conclusions	Reference
**NAM Test System**	**Paired *In Vivo*/Other Data**	**Chemical Characterization**	**Type of Product Tested**	**Sub-classification of Product Tested**	**Extraction Conditions**		
- DPRA<br>- LuSens	NA	NP	Wearables	Consumer products (adult intimate use)	- ISO 10993-12 procedures<br>- Weight:volume ratio - 0.1 g of material:1 mL of DMEM without serum<br>- Conditions: 24 hours; 37°C	- The LuSens assay identified 10 out of the 20 products tested as skin sensitizers, mostly with a silicone, ABS or PVC base.<br>- DPRA identified 3 of the 20 products as sensitizers with a polyisoprene/latex, latex or ABS base, respectively (the products were different from those identified by the LuSens assay).	[103]
Sens-IS	LLNA	NP	Medical devices	Silicone polymers<br><br>(extracts spiked with sensitizers – *see Table S1 in the manuscript for more details*)	- ISO 10993-12 procedures<br>- Weight:volume ratio - 200 mg of polymer:1 mL solvent<br>- Exaggerated condition ratio: weight:volume ratio - 200 mg polymer:0.1 mL solvent<br>- Solvents: polar (0.9% sodium chloride); non-polar (sesame oil)<br>- Conditions: 72 hours; 37°C	- The Sens-IS assay correctly identified sensitizers in 5 of the 6 spiked polymer extracts.<br>- A weak sensitizer (phenyl benzoate) was originally classified as a non-sensitizer; under exaggerated extraction conditions or when using a longer exposure time (6 hours compared to the standard 15 minutes), the assay correctly predicted this chemical as sensitizer.	[106]

Testing Methodologies		Medical Devices and Wearables Specifics				Conclusions	Reference
NAM Test System	Paired *In Vivo*/Other Data	Chemical Characterization	Type of Product Tested	Sub-classification of Product Tested	Extraction Conditions		
- DPRA<br>- LuSens	LLNA	NP	Medical devices	Multiple<br><br>(*see Table S1 in the manuscript for more details*)	- ISO 10993-12 procedures<br>- Surface:volume ratio - 3 cm^2 of sample:1 mL of solvent for solid samples with a defined surface and thickness >0.5 mm<br>- Surface:volume ratio - 6 cm^2 of sample:1 mL of solvent for samples with thickness <0.5 mm<br>- Weight:volume ratio - 0.2 g of sample:1 mL of solvent for samples with undefined surfaces<br>- Solvents: polar (0.9% sodium chloride); non-polar (cottonseed oil)<br>- Conditions: 72 hours; 37°C	- Overall, 1 of the 42 devices tested was predicted as a skin sensitizer in all three test methods (DPRA, LuSens and LLNA).<br>- A total of 33 products were predicted as non-sensitizers.<br>- The combination of LuSens and DPRA could be used to detect low concentrations of sensitizers in medical device extracts, which might be predicted as false negative by LLNA.<br>- DPRA is not suitable for testing metal compounds, as they interact with proteins through non-covalent mechanisms, which may lead to false positive results when present in devices.<br>- A strategy is proposed in which negative results are accepted and any positive results in the DPRA and LuSens assays are to be followed up with a third *in vitro* test (2o3 approach).	[107]
- ADRA<br>- EpiSensA<br>- h-CLAT	- GPMT<br>- LLNA BrdU-ELISA	GC/MS<br>(to quantify DNCB)	Medical devices	Polyurethane (spiked with 1% w/w DNCB)	- ISO 10993-12 procedures	- Regardless of the solvent used, the assays identified the DNCB spiked into the polyurethane sheets as a skin sensitizer.	[108]

Testing Methodologies		Medical Devices and Wearables Specifics				Conclusions	Reference
NAM Test System	Paired *In Vivo*/Other Data	Chemical Characterization	Type of Product Tested	Sub-classification of Product Tested	Extraction Conditions		
					- Solvents: polar (0.9% sodium chloride); non-polar (sesame oil)<br>- Other solvents specific to the respective assays (*see Table S2 in the manuscript for more details*)	- The DNCB-spiked polyurethane sheets can be considered effective extractable positive reference materials for use in testing of medical devices.<br>- The EpiSensA assay, in particular, showed strong sensitivity and may help identify potential sensitizers that are not detected by other *in vitro* methods.	
GARD™skin	LLNA<br><br>(sensitizers used for spiking experiments)	NP	Medical devices	Silicone-based (extracts spiked with sensitizers):<br>- 2-amino-phenol<br>- Cinnamic aldehyde<br>- Phenyl benzoate<br>- Propyl gallate	- ISO 10993-12 procedures<br>- Weight:volume ratio - 0.2 g of product:1 mL of solvent<br>- Solvents: polar (0.9% sodium chloride supplemented with 1% PEST); non-polar (olive oil or sesame oil)<br>- Conditions: 72 hours; 37°C	- The GARD™skin assay was able to correctly classify both polar and non-polar extracts from medical devices that had known sensitization outcomes based *on in vivo* data (*e.g.,* GPMT).<br>- The results reported in this manuscript support the use of the GARD™skin with some adaptations to identify sensitizers spiked in extracts of medical devices.<br>- One of the silicone products spiked with phenyl benzoate and extracted with olive oil generated values of significantly lower magnitude responses compared with extracts prepared in sesame oil and saline, respectively.	[105]
h-CLAT (adaptation)	Consumer-reported	UPLC-MS/MS	Medical & wearable devices	- Watch bands (leather, fabric, silica	- Solvents: neutral (pH 6.5), alkaline (pH 8.0),	- The h-CLAT was successfully adapted for screening of the sensitization potential of consumer skin-contact products and devices.	[7]

Testing Methodologies		Medical Devices and Wearables Specifics				Conclusions	Reference
**NAM Test System**	**Paired *In Vivo*/Other Data**	**Chemical Characterization**	**Type of Product Tested**	**Sub-classification of Product Tested**	**Extraction Conditions**		
	sensitization events			gel, fluoride gel)<br>- Gloves (latex, nitrile, polyvinyl chloride)	and acidic (pH 5.5) artificial sweat<br>- Conditions: 24 hours; 37°C	Methodological adaptations enabled its application to extracts prepared in human artificial sweat.<br>- Neutral and alkaline artificial sweat extracts induced CD54 expression above the positivity threshold for certain products, consistent with consumer reports.<br>- Acidic sweat did not yield concordant results, thus, neutral artificial sweat was considered the preferred extraction solvent for evaluating consumer wearable devices.<br>- While further refinement may enhance its applicability, the proposed adaptation provides a useful foundation for allergy screening of emerging wearable devices.	
- kDPRA<br>- KeratinoSens™	- LLNA (for IBOA, SO60)<br>- GPMT (for ACMO, NNDMA)<br>- HPT (SO60)	NP	Ingredients of Wearables	- Adhesive monomers (ACMO, IBOA, NNDMA)<br>- Dye (SO60)	- Specific to each of the *in vitro* assays used	- Neat constituents of wearable devices can be evaluated for their skin sensitization potential using NAMs, while a PoD is able to be derived in order to conduct a proper risk assessment.<br>- Regression models based on *in vitro* and *in chemico* data were identified, and PoD values were determined for the selected materials that have been linked to have skin sensitization potential.	[104]

Testing Methodologies		Medical Devices and Wearables Specifics				Conclusions	Reference
NAM Test System	Paired *In Vivo*/Other Data	Chemical Characterization	Type of Product Tested	Sub-classification of Product Tested	Extraction Conditions		
						- The results obtained when using the regression model based on kDPRA and KeratinoSens™ made possible to derive pEC3 values (predicted LLNA EC3).	
Sens-IS	LLNA<br>(ISO/TS 11796 Database)	NA	Medical devices	- Solvent-free adhesive (extracts spiked with sensitizers and non-sensitizers – *see Table S1 in the manuscript for more details*)	- ISO 10993-12 procedures<br>- Solvents: polar (physiological saline); non-polar (sesame oil)<br>- Conditions: 72 hours; 50°C	- The Sens-IS assay correctly predicted the sensitization potential of 20 out of 22 chemicals identified as sensitizers in the LLNA, when spiked into medical device polar and non-polar extracts at their respective LLNA EC3 concentrations.<br>- The two mispredicted sensitizers were not soluble at their EC3 concentrations, indicating that solubility limitations may have contributed to the inaccurate results.<br>- While eight non-sensitizers were considered correctly predicted by the Sens-Is assay, the classification for seven of these relied on results from a single extract, due to solubility limitations. This highlights the need to account for solubility constraints when interpreting assay performance.	[109]

2o3, 2 out of 3 approach; ; ABS, Acrylonitrile Butadiene Styrene; ACMO, Acryloylmorpholine; ADRA, Amino Acid Derivative Reactivity Assay; BrdU, Bromodeoxyuridine; CD54, Cluster of Differentiation 54; DMEM, Dulbecco's Modified Eagle Medium; DNCB, 2,4-dinitrochlorobenzene; DPRA, Direct Peptide Reactivity Assay; EC3 value, the amount of a chemical required to elicit a three-fold increase in LLNA; ELISA, Enzyme-Linked Immunosorbent Assay; GARD, Genomic Allergen Rapid Detection; GC/MS, Gas Chromatography/Mass Spectrometry; GPMT, Guinea Pig Maximization Test; h-CLAT, human Cell Line Activation Test; HPT, Human Patch Test; IBOA, Isobornyl acrylate; ISO, International Standards Organization; kDPRA, kinetic Direct Peptide Reactivity Assay; LLNA, Local Lymph Node Assay; NA, Not Applicable; NAM, New Approach Methodology; NNDMA, N, N-Dimethylacrylamide; NP, Not Provided;

pEC3, predicted dose in weight percent that would give a stimulation index value of 3.0 which is regarded as the threshold for positive sensitization; PEST, Penicillin-Streptomycin Solution; PoD, Point of Departure; PVC, Polyvinyl Chloride; SO, Solvent Orange; UPLC-MS/MS, ultrahigh-performance liquid chromatograph coupled with a tandem mass spectrometer.

Note: The references are presented in chronological order and alphabetically within the same year (where applicable).
