# Supplementary material for: New Horizons in Skin Sensitization Assessment of Complex Mixtures: The Use of New Approach Methodologies Beyond Regulatory Approaches
Source: Toxics. 2025 Aug 20;13(8):693. doi: 10.3390/toxics13080693 (PMC12390330; doi:10.3390/toxics13080693)
Supplement: Supplementary file 1 [file toxics-13-00693-s001.zip › Table S4.pdf]

Table S4. New Approach Methodologies used to assess the skin sensitization potential of agrochemicals.

Testing Methodologies		Agrochemicals Specifics	Conclusions	Reference
NAM Test System	Paired *In Vivo*/Other Data	Type of Product Tested		
KeratinoSens™	- Buehler test<br>- GPMT<br>- LLNA	8 agrochemical active ingredients of which:<br>- 3 sensitizers (acetochlor, meptyldinocarp, triclopyr) and 5 non-sensitizers (aminopyralid, clopyralid, forasulam, methoxyfenozide, oxyfluorfen)<br>- 6 herbicides and 2 fungicides<br><br>10 agrochemical formulations of which:<br>- 4 sensitizers, 4 non-sensitizers and 2 equivocal<br>- 8 herbicides, 2 fungicides<br>- 3 were soluble concentrates, 5 emulsion concentrates and 2 suspension concentrates	- All 8 tested agrochemical active ingredients were predicted correctly when compared with *in vivo* data.<br>- The standard KeratinoSens™ method assumed that agrochemical formulations had a common molecular weight. The assay predicted correctly the sensitization potential of 3 out of the 4 sensitizing formulations, and all 6 non-sensitizing formulations when compared with *in vivo* data. This approach is consistent with OECD TG 442D for the KeratinoSens™ assay to test chemicals that have no defined molecular weight.	[111]
- mDPRA<br>- photo-mDPRA	Human predictions (patch tests)	- Glyphosate<br>- POEA<br>- 6 Glyphosate-based herbicides	- mDPRA was conducted using a 10-fold reduction in the reaction volume compared to the conventional DPRA.<br>- Peptide depletion was greater in photo-mDPRA, thereby changing the reactivity class of each test material, in comparison to mDPRA; thus, the association of mDPRA with photo-mDPRA was better at correctly characterizing human sensitizer substances and herbicide formulations that are activated by light.	[112]

Testing Methodologies		Agrochemicals Specifics	Conclusions	Reference
NAM Test System	Paired *In Vivo*/Other Data	Type of Product Tested		
			- For mDPRA, only 2 formulations were predicted positive. When photo-mDPRA was used, the prediction was more robust and was 100% aligned with available human data.	
GARD™skin	NP	- Glyphosate<br>- Surfactant POEA<br>- 2 commercial glyphosate containing formulations	- The skin sensitization potential of glyphosate, POEA, and two POEA-free formulations was assessed using a human dendritic cell model. Initial predictions were made with the GARD™skin assay, followed by proteomic analysis using mass spectrometry on treated cells.<br>- Glyphosate was classified as non-sensitizer, while formulations containing Glyphosate or POEA were classified as sensitizers by the GARD™skin.<br>- Combining GARD™skin results with proteomic analyses can help clarify the skin sensitization potential of agrochemical substances and enhance understanding of the molecular mechanisms underlying immunotoxic effects.	[48]
EpiSensA	- LLNA<br>- GPMT<br>- WoE<br>- *Existing in silico, in vitro data*	- 10 methacrylate esters (7 sensitizers and 3 non-sensitizers)<br>- 5 silicone based compounds (3 sensitizers and 2 non-sensitizers)<br>- 3 crop protection formulations (1 sensitizer, 2 non-sensitizers)<br>- 2 surfactant mixtures (sensitizers)	- EpiSensA correctly predicted 16 out 20 tested materials. The WoE assessments indicated 14 of the chemicals as sensitizers, while 6 were non-sensitizers based on LLNA and GPMT data.<br>- EpiSensA is considered a better predictor for methacrylate when compared with other *in vitro* assays that were used to investigate this class of compounds.	[113]
- GARD™skin<br>- MUTZ-3 cells-based assay	- LLNA<br>- GPMT<br>- Human data (patch test)	- 3 commercial PPP fungicide formulations (Proline EC 250, Shirlane, Folicur Xpert)<br>- 3 adjuvants: POL, NND (surfactant) and BEN (preservative)<br>- 3 individual active constituents: FLU, PRO, TEB	- All single agricultural chemicals investigated in the manuscript were predicted as skin sensitizers using the GARD™skin and the dendritic cells-based assay.<br>- The skin sensitization potency was also predicted for the selected chemicals and mixtures.<br>- The experiments identified responses to agricultural chemicals and products consistent with the dendritic cell model reacting to chemical exposure with oxidative stress, ER stress, effects on autophagy, and metabolic changes especially related to cholesterol homeostasis. After exposure to certain mixtures, novel proteins or transcripts were differentially expressed and these were not detected for any single constituents, supporting the occurrence	[46]

Testing Methodologies		Agrochemicals Specifics	Conclusions	Reference
NAM Test System	Paired *In Vivo*/Other Data	Type of Product Tested		
			of synergistic effects. This indicates that all chemicals in a PPP can contribute to its toxicity profile, including their skin sensitizing/immunotoxic properties.	
- DPRA<br>- KeratinoSens™<br>- h-CLAT<br>- OECD QSAR Toolbox 4.5	- LLNA<br>- GPMT	27 agrochemical formulations of which:<br>- 12 sensitizers and 15 non-sensitizers<br>- 13 water-based liquids (6 suspension concentrates, 7 soluble liquids) and 14 solvent-based (9 emulsion concentrates, 3 oil dispersions, 3 emulsions in water)	- When compared to historical animal results, balanced accuracy for the DAs for predicting *in vitro* skin sensitization hazard (*i.e.*, sensitizer *vs.* non-sensitizer) ranged from 56 to 78%.<br>- The 2o3 was the best performing DA, in which the hazard classification was based on two concordant results from the DPRA, KeratinoSens™, and/or h-CLAT.<br>- The KE 3/1 STS (based on h-CLAT and DPRA results), and ITSv2 (using h-CLAT, DPRA, and an *in silico* hazard prediction from OECD QSAR Toolbox), had balanced accuracies of 56-57% for hazard classification.<br>- Of the individual test methods, KeratinoSens™ had the best performance for predicting *in vivo* hazard outcomes. Its balanced accuracy of 81% was similar to that of the 2o3 DA (78%).<br>- For predicting potency categories defined by the United Nations Globally Harmonized System of Classification and Labelling of Chemicals, the correct classification rate of the STS was 52% and that of the ITSv2 was 43%.	[5]
- GARD™skin<br>- GARD™potency	- Buehler Test<br>- LLNA<br>- Magnusson & Kligman test	Agrochemical formulations<br>- 15 liquid (water-based)<br>- 15 liquid (solvent-based)<br>- 12 solid	- Both GARD™skin and GARD™potency performed satisfactorily when used on a representative set of 42 agrochemical formulations.<br>- GARD™skin demonstrated an overall accuracy of 76.2%, sensitivity of 85.0%, and specificity of 68.2% compared to historical animal data.<br>- Most misclassifications could be attributed to borderline results in the animal data or low-level presence of sensitizers in the formulations.<br>- GARD™potency was able to correctly subcategorize 14 out of 17 sensitizers by GHS category, with only a few over- or under-predictions.	[114]

2o3, 2 out of 3 approach; BEN, 1,2-benzisothiazol-3(2 H)-one; DA, Defined Approach; DPRA, Direct Peptide Reactivity Assay; ER, Endoplasmic Reticulum; FLU, fluazinam; GARD, Genomic Allergen Rapid Detection; GHS, Globally Harmonized System; GPMT, Guinea Pig Maximization Test; h-CLAT, human Cell Line Activation Test; ITS, Integrated Testing Strategy; KE, Key Event; LLNA, Local Lymph Node Assay; mDPRA, micro or modified DPRA; NAM, New Approach Methodology; NND, N,N-dimethylcapramide; NP, Not Provided; OECD, Organization for Economic Co-operation and Development; photo-mDPRA, phototoxicity-adapted modified Direct Peptide Reactivity Assay; POEA, polyoxyethylene tallow amine; POL, poly(oxy-1,2- ethanediyl), alpha-sulfo-omega-[2,4,6-tris(1-phenylethyl)phenoxy]-, ammonium salt; PPP, Plant Protection Products; PRO, prothioconazole; QSAR, Quantitative Structure-Activity Relationship; STS, Sequential Testing Strategy; TEB, tebuconazole; TG, Test Guideline; WoE, Weight of Evidence.

Note: The references are presented in chronological order and alphabetically within the same year (where applicable).
