# Supplementary material for: New Horizons in Skin Sensitization Assessment of Complex Mixtures: The Use of New Approach Methodologies Beyond Regulatory Approaches
Source: Toxics. 2025 Aug 20;13(8):693. doi: 10.3390/toxics13080693 (PMC12390330; doi:10.3390/toxics13080693)
Supplement: Supplementary file 1 [file toxics-13-00693-s001.zip › Table S5.pdf]

Table S5. New Approach Methodologies used to assess the skin sensitization potential of pollutants.

Testing Methodologies/NAM Test System	Type of Pollutant*	Sub-classification of Product Tested	Chemical Characterization	Sampling Procedure	Conclusions	Reference
- GARD™skin<br>- GARDpotency Assay	Tobacco	e-liquids:<br>- 3 experimental base liquids<br>- 2 commercial	NP	NA	- The base liquids with or without nicotine were classified as non-sensitizers, which is in agreement with expected potential based on the composition.<br>- From the other components with otherwise known toxicity profiles, nicotine and polypropylene glycol were not predicted as sensitizers.<br>- The two commercial e-liquids were classified as potential skin sensitizers and GHS Cat 1B (weak sensitizers).<br>- GARD assays were able to differentiate and broadly classify e-liquids (which are considered mixtures) based on their sensitizing potentials.	[151]
- DPRA<br>- LuSens	Indoor air condensate	- VOCs (25)<br>- Genapol X-80 (nonionic emulsifier)	For VOCs:<br>- GC/FID<br>- GC/ECD<br><br>For Genapol X-80:<br>- HPLC<br>- HPLC-MS	40 water samples condensed from indoor air were collected from different facilities in Finland, including private homes, public buildings,	- Indoor air is considered a mixture.<br>- VOCs and Genapol X-80 were not detected in indoor air samples, however, sensitization potential was detected.<br>- Of the 39 samples tested using the DPRA, 26 were predicted to be sensitizers, while 13 were predicted non-sensitizers. One typical DPRA-inducer is formaldehyde, which is commonly present in indoor as well as outdoor environments.<br>- Of the 40 samples tested using the LuSens assay, 8 were predicted to be skin sensitizers and the rest non-sensitizers.	[152]

Testing Methodologies/NAM Test System	Type of Pollutant*	Sub-classification of Product Tested	Chemical Characterization	Sampling Procedure	Conclusions	Reference
				offices and schools, using stainless steel collectors (39 were tested for skin sensitization)	- Of the samples tested with both the DPRA and LuSens assays, seven were predicted as positive by both methods. Five of these were from the 25 percent condensate samples and two from the 50 percent condensate samples.<br>- Analyzing individual chemicals is not an adequate approach to assess indoor air pollution. The NAMs were able to identify skin sensitizers.	

Cat, Category; DPRA, Direct Peptide Reactivity Assay; GARD, Genomic Allergen Rapid Detection; GC/ECD, Gas Chromatography with Electron Capture Detection; GC/FID, Gas Chromatography with Flame-Ionization Detection; HPLC, High-Performance Liquid Chromatography; GHS, Global Harmonized System; MS, Mass Spectrometry; NA, Not Applicable; NAM, New Approach Methodology; NP, Not Provided; VOC, Volatile Organic Compounds.

* - No comparative *in vivo* data reported for any of the manuscripts.

Note: The references are presented in chronological order and alphabetically within the same year (where applicable).
